# Mechanisms of Hexavalent Chromium-Induced Lung Carcinogenesis: DNA Double-Strand Breaks and DNA Repair Inhibition in a Guinea Pig Model

**DOI:** 10.3390/ijms27198529

**Published:** 2026-09-24

**Authors:** Idoia Meaza, Haiyan Lu, Jamie L. Wise, Sandra Diven, Aggie W. Brownell, John Easley, J. Calvin Kouokam, John P. Wise, Samuel T. Vielee, James T. F. Wise, John Pierce Wise

**Affiliations:** 1 Wise Laboratory of Environmental and Genetic Toxicology, Department of Pharmacology and Toxicology, University of Louisville, Louisville, KY 40202, USA; idoia.isusi@louisville.edu (I.M.);; 2 Wise Laboratory of Liver and Environmental Health, Department of Pharmacology and Toxicology, University of Louisville, Louisville, KY 40202, USA; 3 NeuroWise Laboratory of Environmental Toxicology, University of Toledo, Toledo, OH 43614, USA; 4 Department of Pharmacology and Toxicology, University of Louisville, Louisville, KY 40202, USA; 5 Mote Marine Laboratory, Sarasota, FL 34236, USA; 6 Wise Laboratory of Nutritional Toxicology and Metabolism, University of Hawaii, Honolulu, HI 96822, USA

**Keywords:** hexavalent chromium, lung carcinogenesis, metals, DNA damage, DNA repair, RAD51, homologous recombination repair, DNA double-strand breaks, guinea pigs, oropharyngeal aspiration

## Abstract

Hexavalent chromium [Cr(VI)] is an environmental and occupational pollutant and a known lung carcinogen. Despite the exposure risks, it is unclear how Cr(VI) causes lung cancer. Prolonged Cr(VI) exposure induces DNA double-strand breaks and inhibits their repair in exposed cells, leading to chromosome instability, a hallmark of lung cancer. Specifically, Cr(VI) inhibits RAD51, a key effector protein in the homologous recombination repair pathway. The goal of this study was to translate this molecular mechanism of Cr(VI)-induced lung carcinogenesis to a guinea pig model. Hartley guinea pigs 10 to 11-week-old were exposed to zinc chromate via oropharyngeal aspiration for acute (24 h) or subchronic (90-day) exposure. Lungs were dissected and formalin-fixed paraffin-embedded. DNA double-strand breaks were measured using gamma-H2AX foci, and homologous recombination repair was assessed using RAD51 foci. Acute Cr(VI) exposure induced gamma-H2AX foci and RAD51 foci, whereas subchronic exposure also caused gamma-H2AX foci but inhibited RAD51 foci. These effects of Cr(VI)-induced DNA damage and repair inhibition were largely observed in bronchiolar regions but not in alveolar regions. This study establishes a new human-relevant model to study Cr(VI)-induced carcinogenesis and translates the mechanism of Cr(VI)-induced DNA double-strand break formation and repair inhibition to guinea pig lungs.

## 1. Introduction

Lung cancer is a major public health concern as it is the number one cause of cancer death worldwide [1]. The 5-year survival rate for lung cancer is 28% and has not dramatically improved for decades [2]. This lack of progress in improving the survival rates is likely fueled by the misperception that lung cancer is just caused by smoking and that the cessation of smoking can solve this problem. However, lung cancer in never-smokers is still the fifth leading cause of cancer death, which suggests a relevant contribution of other risk factors to this disease [3]. Indeed, a group of major lung carcinogens includes metals. Particularly, there are five metals, including nickel, cadmium, arsenic, beryllium and hexavalent chromium [Cr(VI)], that are known human lung carcinogens and are among the eight most dangerous lung carcinogens in occupational settings [4,5].

Cr(VI) is of special concern due to its potency as a human lung carcinogen and a widespread environmental and occupational pollutant [6,7,8]. Despite it being a public health hazard and raising concerns due to its cumulative nature, meaning a low-dose exposure over many years can cause the same disease as a high dose in an occupational setting, how Cr(VI) causes lung cancer is still unclear. Most of the studies assessing the molecular mechanisms behind Cr(VI)’s carcinogenic process suggest DNA damage and chromosome instability are central to developing neoplastic transformation and malignancy [9]. Specifically, Cr(VI) causes DNA double-strand breaks and inhibits their repair by targeting RAD51 in homologous recombination repair, leading to the progression of these breaks to chromosome instability [10,11,12,13]. The latter is known to be a hallmark of lung cancer and a determining step for Cr(VI)-induced neoplastic transformation [14].

While there are some studies assessing Cr(VI) carcinogenicity in rodent models, translation to humans has been extremely limited due to major physiological and morphological differences in the lungs between the rodent models used and humans. This study focuses on translating the mechanism of Cr(VI)-induced DNA double-strand breaks and repair inhibition to guinea pigs because, unlike mice or rats, they have a dichotomous bronchiole bifurcation pattern, which is the same pattern observed in human lungs [15]. The bronchiole pattern is a crucial consideration to assess Cr(VI) carcinogenicity as it can directly affect particle deposition. Based on particle physics and pathological studies in chromate workers who developed lung tumors, Cr(VI) particles impact at bronchial bifurcations, where they slowly dissolve, releasing Cr(VI) and forming tumors at these sites [16,17,18]. In terms of lung anatomy, mice and rats have monopodial bifurcations, which could allow deeper penetration of particles into the lungs and higher alveolar deposition. In contrast, a dichotomous branching pattern increases the probability of particles impacting bronchiolar bifurcations.

Another important consideration for using guinea pigs for Cr(VI) inhalation studies is that rodents like mice and rats produce their own ascorbic acid (vitamin C) in their livers [15]. On the contrary, humans and guinea pigs do not, and require vitamin C intake from their diet. The production of vitamin C (or lack thereof) is an important difference that affects lung susceptibility to Cr(VI), as studies have shown a 7-to 9-fold increase in vitamin C levels in rat lung bronchoalveolar fluid compared to levels in guinea pigs and humans [19]. Levels of vitamin C in the lung, particularly in the extracellular matrix, may be important for Cr(VI) effects, as it is one of the key reductants of Cr(VI). When Cr(VI) is reduced extracellularly to Cr(III), the trivalent form tends to bind to other ligands, making it too bulky to pass cell membranes and thus drastically decreasing the uptake into the cells, which is a necessary step to exert its carcinogenic potential. Thus, studying the effects of Cr(VI) in mice or rats, which have higher levels of vitamin C in the lungs, could lead to an underrepresentation of Cr(VI)’s toxicological effects.

Given the importance of choosing a rodent model that simulates human lung morphology and physiology, we chose a guinea pig model. This study aimed to translate the mechanism of Cr(VI)-induced DNA double-strand breaks and inhibition of repair in guinea pig lungs and to develop a new model to study Cr(VI) carcinogenesis for future mechanistic studies. Thus, we hypothesize acute exposure to guinea pigs will induce DNA repair in lung cells, while subchronic exposure will inhibit DNA repair in lung cells.

## 2. Results

### 2.1. Acute Exposure to Cr(VI) Induces DNA Double-Strand Breaks in Guinea Pig Lungs

It has been well-established that Cr(VI) exposure induces DNA double-strand breaks and these are the preceding lesions to chromosome instability in Cr(VI)’s carcinogenic mechanism. When DNA double-strand breaks occur, the Ser139 of the histone H2AX is phosphorylated, turning it into gamma-H2AX (γH2AX). The number of phosphorylated γH2AX foci at DNA double-strand break sites represents the number of breaks [20,21,22]. Thus, we quantified Cr(VI)-induced DNA double-strand breaks using γH2AX in oropharyngeal-exposed guinea pig lungs. Further, given that previous pathology studies show Cr(VI)-induced tumors arise from lung epithelial cells, we used cytokeratin to only consider epithelial cells in our analyses. Our results showed acute oropharyngeal Cr(VI) exposure-induced DNA double-strand breaks in epithelial cells of guinea pig lungs (Figure 1, Figure 2 and Figure S1). Specifically, acute exposure to saline, 0.2, 0.4 and 0.8 mg/kg zinc chromate led to 948.8, 1282.5, 1212.7 and 1092.6 total γH2AX foci in 100 bronchiolar cells, respectively, and 271.7, 366.6, 329.4 and 306.9 total γH2AX foci in 100 alveolar cells, respectively. While only the lowest dose (0.2 mg/kg zinc chromate) induced a significant increase in DNA double-strand breaks compared to controls, the middle dose (0.4 mg/kg zinc chromate) approached significance in the bronchioles (*p* = 0.1433).

We considered the effects in males and females separately and observed similar trends. For example, in the males, acute exposure to saline, 0.2, 0.4 and 0.8 mg/kg zinc chromate caused 1097.7, 1594.3, 1502.6 and 1240.6 total γH2AX foci in 100 bronchiolar cells, respectively, while in the females there were 799.9, 970.7, 922.9 and 944.7 total γH2AX foci in 100 bronchiolar cells, respectively. In the alveoli, acute exposure to saline, 0.2, 0.4 and 0.8 mg/kg zinc chromate led to 336.3, 450.3, 446.7 and 396.6 total γH2AX foci in 100 cells in males and 207.1, 282.9, 212 and 217.3 total γH2AX foci in 100 cells in females. Comparing the levels in males and females, males had overall higher levels of DNA double-strand breaks.

### 2.2. Acute Exposure to Cr(VI) Induces DNA Double-Strand Break Repair in Bronchiolar Epithelial Cells

Cr(VI)-induced DNA double-strand breaks can be repaired through homologous recombination repair. Indeed, previous data from our laboratory and others showed that acute Cr(VI) exposure induces homologous recombination repair, observed by an increase in RAD51 foci [10,12]. RAD51 is a key protein in homologous recombination repair that binds to single-stranded DNA and forms nuclear filaments that guide the strand exchange and homology search [23]. Thus, to determine whether acute exposure to Cr(VI) activated homologous recombination repair in guinea pig lungs, we assessed the levels of RAD51 foci. We found acute exposure to zinc chromate induced RAD51 foci levels in the bronchioles but not in the alveoli (Figure 3, Figure 4 and Figure S2). Specifically, acute exposure to saline, 0.2, 0.4 and 0.8 mg/kg zinc chromate led to 177.1, 369.9, 379.2 and 259.9 total RAD51 foci in 100 bronchiolar cells, while causing 135.0, 197.6, 194.3 and 83.9 total RAD51 foci in 100 alveolar cells, respectively. The low and middle doses of zinc chromate induced a statistically significant increase in RAD51 levels in the bronchioles but not in the alveoli, and the highest dose in the bronchioles almost induced a significant increase in RAD51 (*p* = 0.0519). No sex-dependent differences were observed for the RAD51 response; however, when separating the data by sex, we observed overall higher levels in the males for the low and middle doses. Specifically, the middle dose in the males almost induced a significant increase in the RAD51 (*q* = 0.0787).

### 2.3. Acute Exposure to Zinc Chromate Increases Repair, Particularly in the Bronchioles

To directly analyze the outcomes observed for DNA double-strand breaks and DNA repair in guinea pigs after acute zinc chromate exposure, we used a DNA double-strand break repair ratio. For this ratio, the levels of DNA double-strand breaks (total γH2AX foci) in an animal are divided by the levels of repair (total RAD51 foci) in the same animal. A decrease in this ratio shows an increase in DNA repair, indicated by a decrease in DNA breaks or an increase in RAD51 levels. On the contrary, an increase in the ratio indicates an inhibition of repair, as the number of DNA breaks increases relative to RAD51 levels. Importantly, previous studies in the field of DNA repair have also used this γH2AX/RAD51 ratio as well as other DNA repair protein ratios, such as the γH2AX/MRE11 ratio, as an indicator of overall DNA repair [24,25]. An increase in the γH2AX/RAD51 ratio reflected a deficiency in homologous recombination repair and this effect was confirmed in BRCA1-mutated cell lines with deficient repair [25].

Our results showed the DNA double-strand break repair ratio decreased after acute Cr(VI) exposure in the bronchioles, suggesting DNA double-strand break repair was active (Figure 5). Specifically, acute exposure to saline, 0.2, 0.4 and 0.8 mg/kg zinc chromate led to 9.4, 6.6, 4.6 and 6.3 γH2AX/RAD51 DNA repair ratios, respectively, in the bronchioles, while they induced 3.9, 3.5, 3.1 and 7.1 γH2AX/RAD51 DNA repair ratios in the alveoli. Of these conditions, only the middle dose induced a significant decrease in the bronchioles compared to saline controls, although the lowest dose almost achieved significance (*p* = 0.1277). No sex-dependent differences were observed between males and females in the bronchioles, although the lowest DNA repair ratios were observed in the females, which indicated enhanced repair. In the alveoli, the highest dose of zinc chromate induced a higher DNA repair ratio in the males, and this effect almost achieved significance (*q* = 0.0735).

### 2.4. Subchronic Exposure to Cr(VI) Causes DNA Double-Strand Breaks in Bronchiolar Epithelial Cells

To determine whether zinc chromate exposure induces DNA double-strand breaks after subchronic exposure, we measured γH2AX foci in bronchiolar and alveolar epithelial cells. We found after subchronic exposure, zinc chromate significantly induced DNA double-strand breaks in the bronchioles but not in the alveoli (Figure 6, Figure 7 and Figure S3). Specifically, subchronic exposure to saline, 0.2, 0.4 and 0.8 mg/kg zinc chromate induced 800.5, 1163.4, 1139.7 and 1191.1 total γH2AX foci in 100 bronchiolar cells, and 367.6, 338.4, 371.6 and 374.2 total γH2AX foci in 100 alveolar cells, respectively. All zinc chromate doses induced significant levels of DNA double-strand breaks in the bronchioles.

When considering males and females separately, we did not observe significant sex-dependent differences in the bronchioles. However, overall, females had higher levels of DNA double-strand breaks in the highest two doses in the bronchioles. In the alveoli, this trend was also found, and the highest dose of zinc chromate almost achieved significance (*q* = 0.058).

### 2.5. Subchronic Exposure to Cr(VI) Inhibits DNA Double-Strand Break Repair in the Bronchioles

While we observed an increase in RAD51 after acute exposures to zinc chromate, previous studies have shown prolonged exposures to Cr(VI) inhibit homologous recombination repair through the targeting of RAD51 [11,26,27]. Thus, we assessed whether subchronic exposure to zinc chromate causes loss of RAD51 foci in guinea pig lung cells. Contrary to the response observed after acute exposure, we indeed found subchronic exposure inhibited RAD51 levels (Figure 8, Figure 9 and Figure S4). Specifically, subchronic exposure to saline, 0.2, 0.4 and 0.8 mg/kg zinc chromate led to 162.6, 130.9, 134.1 and 130.1 RAD51 foci in 100 bronchiolar cells and 96.1, 78.1, 81.6 and 79.7 RAD51 foci in 100 alveolar cells, respectively. When considering males and females separately, we observed that in males the middle dose induced a decrease in foci levels and this effect almost achieved significance (*q* = 0.1181), while in females, RAD51 foci levels remained similar to those of controls. For example, in the bronchioles, saline, 0.2, 0.4 and 0.8 mg/kg zinc chromate caused 172.4, 108.7, 88.6 and 120.9 RAD51 foci in 100 cells in male guinea pigs, while inducing 152.9, 153.0, 179.6 and 139.3 RAD51 foci in 100 cells in females, respectively. This effect between the sexes was also observed in the alveoli.

### 2.6. Subchronic Cr(VI) Exposure Inhibits DNA Repair in Guinea Pig Lungs

The DNA repair ratio was also calculated to better understand the DNA repair status after subchronic exposure of guinea pigs to zinc chromate. The ratio was calculated by dividing the number of γH2AX foci by the number of RAD51 foci in each animal. Our results showed the DNA repair ratio significantly increased in the bronchioles of Cr(VI)-exposed animals, which is indicative of a decreased DNA repair capacity (Figure 10). Specifically, subchronic exposure to saline, 0.2, 0.4 and 0.8 mg/kg zinc chromate led to 6.0, 11.6, 14.5 and 12.1 γH2AX/RAD51 DNA repair ratios in the bronchioles and 5.3, 5.8, 7.9 and 6.2 γH2AX/RAD51 DNA repair ratios in the alveoli, respectively. The increase in the DNA repair ratio in the bronchioles was significant for all concentrations. The inhibition of DNA repair was similar across both sexes, with males having a slightly higher degree of inhibition. For example, in the bronchioles, exposure to saline, 0.2, 0.4 and 0.8 mg/kg zinc chromate caused 6.2, 14.3, 16.4 and 12.1 γH2AX/RAD51 DNA repair ratios in males and 5.8, 9.3, 13.0 and 12.0 γH2AX/RAD51 DNA repair ratios in females, respectively. Indeed, the first two doses of zinc chromate almost induced a significant increase in the DNA repair ratio (*q* = 0.1389 and *q* = 0.1272, respectively). A similar trend across the sexes was also observed in the alveoli.

## 3. Discussion

Cr(VI) is a well-known human lung carcinogen, yet how it causes this disease is still unclear. Among the best-characterized steps in its carcinogenic mechanism are DNA double-strand breaks and inhibition of their repair through targeting of RAD51 [9]. In addition, from pathology data in chromate workers, we know Cr(VI)-induced tumors arise from epithelial cells and bronchial bifurcations are a hotspot for particle deposition [16,17,18,28]. Given the importance of lung bifurcations in Cr(VI)’s carcinogenic mechanism, it is crucial to investigate whether the mechanism of Cr(VI)-induced DNA breaks and repair inhibition translates to a rodent model with similar lung anatomy and physiology as humans. This is the first study to address this major data gap and translate the carcinogenic mechanism of Cr(VI) to guinea pig lungs. Guinea pigs are a relevant model for Cr(VI) because (1) they have similar lung bifurcations as humans, which can affect particle deposition and (2) they have similar levels of vitamin C in the lungs, which is a major reductant of Cr(VI) and thus can affect Cr(VI) toxicity [15,19]. Our results show Cr(VI) induces DNA double-strand breaks after acute and subchronic exposures, but only activates DNA repair after acute exposure, while inhibiting it after subchronic exposure, which is consistent with previous reports.

Acute exposure to Cr(VI) induces DNA double-strand breaks and their repair, observed as an increase in RAD51 foci levels, leading to a decreased DNA repair ratio, which indicates repair is active, corroborating many previous studies in cultured cells [10,12,29,30,31,32,33,34,35]. In addition, our results are consistent with a previous study assessing DNA double-strand breaks and repair in Wistar rats exposed to zinc chromate through oropharyngeal aspiration [10]. While all zinc chromate doses caused increases in the levels of DNA double-strand breaks, the lowest dose caused the most significant increase. One possibility is that the intracellular Cr levels are too high after exposure to the middle and highest doses and the cells die, which is also consistent with the higher RAD51 levels detected at the low dose following that peak in breaks. This is also supported by the higher levels of Cr observed in the middle and highest doses in acutely exposed guinea pigs [36]. Cr(VI)’s effects strongly correlate with intracellular Cr levels rather than the exposure concentration, and an excessive sudden Cr accumulation at higher doses may eliminate the most severely damaged cells from the epithelium. However, whether this occurs in Cr(VI)-exposed guinea pig lungs remains to be tested.

Subchronic exposure to Cr(VI) induced DNA double-strand breaks but inhibited repair in the bronchioles. Previous cell culture studies showed prolonged Cr(VI) exposure (1) inhibits RAD51 repair foci, (2) reduces nuclear localization and increases aberrant cytoplasmic localization, (3) reduces RAD51 RNA levels, (4) decreases protein levels, and (5) inhibits homologous recombination [12,27,37,38]. This is the first study to translate inhibition of RAD51 foci formation to a guinea pig model exposed to Cr(VI) particles through oropharyngeal aspiration and is consistent with a previous study showing RAD51 inhibition in tumor tissues from chromate workers and in Cr(VI)-exposed Wistar rats [11]. These results are also consistent with the higher levels of methylation of RAD51 CpG sites in blood samples of chromate workers compared to controls, which suggests transcriptional inhibition of this repair gene with Cr(VI) exposure [39]. Further, cell culture studies have also confirmed that loss of homologous recombination repair is a heritable and persistent phenotype often observed in Cr(VI)-transformed cells [14]. Together, these studies and ours suggest Cr(VI)-induced inhibition of homologous recombination repair occurs after prolonged exposures and is a key event in the mechanism of Cr(VI) carcinogenesis.

Measurements of γH2AX itself may also reflect replication stress, apoptosis, oxidative stress, or other forms of cellular injury. However, γH2AX foci are widely used and accepted by the field of DNA damage and repair as a marker for DNA double-strand breaks [40,41,42,43,44]. Indeed, previous studies have shown that the number of γH2AX foci correlates with the number of DNA double-strand breaks [45]. The important distinction is the specific measurement of the foci. For example, γH2AX involved in apoptosis measured with immunofluorescence assays typically manifests as an apoptosis ring signal around the nucleus, and these foci did not manifest in our analysis and if they had would not have been counted in our analysis, as we only considered distinct foci in the nucleus [46]. Similarly, other studies show γH2AX involved in replication stress in the nucleus manifests as a pan-nuclear γH2AX pattern rather than localized foci [47]. In addition, the robust literature shows Cr(VI) is a strong inducer of DNA double-strand breaks, and the increase in Cr(VI)-induced γH2AX foci is simultaneously observed with an increase in DNA double-strand breaks measured by other assays such as the comet assay, or other immunofluorescence assays. Thus, while γH2AX might also be involved in other pathways, given their difference in staining patterns compared to the repair foci and the existing literature showing Cr(VI) induces DNA double-strand breaks, we believe the γH2AX foci we observed are DNA double-strand breaks, which is consistent with the presentation of these foci in the DNA damage and repair fields.

Cr(VI)-induced lung cancers are mostly squamous cell carcinomas, which are known to arise from epithelial cells [48,49]. Most of the effects, particularly after subchronic exposure, occurred in bronchiolar epithelial cells and not in alveoli. Other studies have also determined that inhaled Cr(VI) particles impact bronchial bifurcations, creating Cr hotspots, where they induce tumors in workers, suggesting particles do not reach the alveoli, consistent with the lack of effects in the alveoli observed in this study [16,17,18]. These studies, together with other studies assessing the carcinogenicity of Cr(VI) particles in rats via intrabronchial implantation, show Cr(VI) carcinogenesis occurs in the bronchiolar epithelium and not the alveoli, and particulates are the most potent Cr(VI) carcinogens [28,50]. Although some effects of Cr(VI) were indeed observed in the alveoli, particularly after acute exposure, where DNA double-strand breaks slightly increased, this effect is likely due to a small number of smaller Cr(VI) particles managing to reach the alveoli. However, by subchronic exposure, the levels of DNA double-strand breaks remain closer to those of the controls, indicating that they were repaired. Altogether, our data suggest the bronchiolar epithelium is most susceptible to Cr(VI)-induced induction of DNA double-strand breaks and inhibition of homologous recombination repair.

Males appeared to have worse DNA repair than females, particularly in the low and middle doses. Consistent with our results, there are a few studies that considered sex in cancer rates and mortality and reported that men are at a higher risk of lung cancer than women [51,52,53]. For example, lung cancer mortality rates among chrome platers in the United Kingdom were higher in males than in females [51]. However, studies assessing sex-dependent effects in Cr(VI) carcinogenicity and mortality are scarce, as early epidemiological studies focused on male workers, because chromate industries were male-dominated occupations. However, males may be more susceptible to Cr(VI) carcinogenicity, as some studies have shown women had more efficient mechanisms of DNA repair and less overall oxidative stress [54,55]. Further, others have shown that estrogens and progesterone in females can activate p53 while male sex hormones like testosterone reduce p53 expression [56,57,58]. For example, in breast cancer, estrogens positively regulate the expression of NBS1, which is a key component of the MRN complex responsible for the initial signaling of DNA double-strand breaks. [59]. Thus, females may be slightly protected against Cr(VI)-induced DNA double-strand breaks.

Lastly, we would like to discuss some of the limitations of this study. The mechanism of Cr(VI)-induced carcinogenesis is a complex process with many steps that have been established using cell culture models. This study does not translate all the carcinogenic steps to animal tissues since it would require an unethical number of animals, but rather translates the two most important events, the induction of DNA double-strand breaks by Cr(VI) and the inhibition of homologous recombination repair. It might be possible that the decrease in RAD51 foci after 90-day exposure to zinc chromate in the bronchioles results from altered cell proliferation, selective loss of damaged cells, or increased cytotoxicity, rather than an inhibition of homologous recombination repair. However, these alternative explanations have not been demonstrated in vivo for Cr(VI). Moreover, extensive data from cell culture studies by our laboratory and others show prolonged Cr(VI) exposure inhibits homologous recombination repair by targeting RAD51 [12,26,37,38,60,61]. Cr(VI) exposure disrupts RAD51 filament formation and induces aberrant cytoplasmic accumulation, leading to a decrease in sister chromatid exchanges and homology-directed DNA repair, measured through a reporter assay [12,26,37,38,60,61]. In addition, Cr(VI)-exposed cells that survive after exposure show chromosomal aberrations and DNA damage, suggesting that the selective loss of damaged cells is unlikely to fully explain the reduction in RAD51 foci observed in guinea pig lung tissues [14]. Thus, based on the available empirical evidence, we believe the interpretation that homologous repair is inhibited by Cr(VI) is a reasonable conclusion.

We also acknowledge that while our stock preparation and dosing methodology produces a consistent size range of particles, it is possible that particles might aggregate in different ways in the lung at bifurcation sites, which are consistent with the site where Cr(VI) causes tumors. However, due to the difficulties in accurately measuring the size of particle aggregates in the lung tissues at locations where the foci are measured, it is uncertain whether this potential aggregation impacts the potency of the Cr(VI) dose. Another potential limitation of the study is that we use an oropharyngeal aspiration method to deliver the zinc chromate particles. However, this method was chosen because from all the types of exposures to the lung, it is the most reliable one for repeated chronic exposures. Particularly, this method results in minimal loss of particles into the gastrointestinal tract, ensuring consistent delivery to the lungs. The alternatives, inhalation and intratracheal instillation methods, have major limitations. Rodents, including guinea pigs, have extensive nasal turbinates which would trap Cr(VI) particles in the passages leading to a higher exposure to the nasal cavity and lower exposure to the lungs in rodents compared to those in humans. Further, intratracheal instillation requires surgical procedures for each dose, which would cause significant distress to animals exposed weekly during subchronic exposures. Altogether, oropharyngeal exposure provides a practicable exposure route to study subchronic effects of Cr(VI).

## 4. Materials and Methods

### 4.1. Animal Husbandry and Experimental Design

Animals were purchased, maintained and exposed to zinc chromate particles as previously described [36]. Fifty-six male and fifty-six female Hartley guinea pigs at 63–69 days (10 to 11 weeks) of age were purchased from Charles River (Wilmington, MA, USA). The guinea pigs were housed at the University of Louisville animal facility under standard laboratory conditions. This animal study was approved by the University of Louisville Institutional Animal Care and Use Committee (Protocol # 23244) and thus, the specified guidelines for animal care and use were applied.

When animals arrived at the animal facility, they were put on a Teklab Global Guinea Pig Diet (2040C, certified) and were allowed to acclimate to the facility and the diet for a week, with water and food ad libitum, before exposures began. The diet was chosen because it meets the essential nutrient needs of guinea pigs and is certified to have low levels of nonessential metals, which is critical for this study to reduce confounding factors. Animals were housed under a 12 h light/dark cycle. To identify the guinea pigs, we implanted a microchip in each animal.

Two exposure times were considered in this study: an acute, single-dose, 24 h exposure and a subchronic, repeated-dose exposure where animals were exposed to zinc chromate particles once a week for 90 days (13 weeks) (Figure 11). Each exposure group (acute and subchronic exposure) consisted of 56 animals (28 males and 28 females). Animals were further divided into groups of 7 and exposed to either saline or zinc chromate particles suspended in saline via oropharyngeal aspiration. Specifically, we considered a low dose (0.2 mg/kg zinc chromate), a middle dose (0.4 mg/kg zinc chromate) and a high dose (0.8 mg/kg zinc chromate). These concentrations are consistent with previous studies of oropharyngeal aspiration of Cr(VI) particles and are below occupational exposures and within environmentally relevant concentrations, as described before [36]. To ensure there was a homogeneous distribution across groups, animals were divided into groups based on their body mass.

### 4.2. Zinc Chromate Particle Preparation

Zinc chromate was selected as the representative particulate Cr(VI) compound, as particulate Cr(VI) compounds are known to be the most potent Cr(VI) carcinogens and have been directly linked to an increase in lung cancer cases in exposed workers [6,8,62,63]. Zinc chromate (ZnCrO4; CAS #13530-65-9) was purchased from Pfaltz and Bauer and the purity was 99.7%. Before preparing the stock solutions, zinc chromate particles were washed twice with water to remove water-soluble contaminants and twice with acetone to remove organic contaminants. Particles were then allowed to dry and were ground with a mortar and pestle and stored until use.

All particle suspensions were prepared the day before each treatment. First, zinc chromate particles were weighed and added to a scintillation vial with a stir bar. Then, cold filter-sterile water was added, and the suspension was stirred overnight at 4 °C to ensure homogeneous particle size distribution. Using this method, zinc chromate particles ranged from 1.8 to 2.6 µm in diameter, with an average particle diameter of 2.2 µm determined by dynamic light scattering [13]. The following day, dilutions were made using cold sterile normal saline [0.9% sodium chloride purchased from Cytiva Life Sciences (Marlborough, MA, USA)] while continuously vortexing to avoid particles settling in the bottom of the vial.

### 4.3. Oropharyngeal Aspiration Exposure

Animals were exposed to zinc chromate particles through oropharyngeal aspiration, as previously described [36,64]. Briefly, under a chemical fume hood, one animal at a time was placed in an animal chamber with continuous oxygen flow and 4% isoflurane anesthesia. After anesthesia, the animals were gently taken out of the chamber under the chemical hood and were placed in a supine position at an angle. The nose was then gently blocked and an appropriate volume of zinc chromate solution or saline was pipetted into the back of the throat. The breathing of the guinea pigs was then monitored to ensure they took at least 15 breaths through the mouth or until they woke up. The total volume administered did not exceed 50 μl per dose. Once in their cage, animals were monitored to ensure proper recovery and normal function.

Throughout the study, humane endpoints were in place to minimize pain or distress. The weight was monitored every week before the animals were exposed and if the animals experienced a 15% weight loss or pronounced lethargy, labored breathing, or problems with movement, so as to produce difficulty with eating or drinking regardless of weight status, euthanasia of these animals was considered with the veterinarian at the animal facility. The animals were monitored daily in the animal facility, anesthesia was provided for all treatments, and the volume of zinc chromate was limited to 50 μl. At the end of the study appropriate euthanasia methods were used as described below.

### 4.4. Organ Collection and Tissue Processing

At the end of exposure periods, animals were anesthetized with a lethal dose of 120 mg/kg ketamine and xylazine (1:12 ratio). Toe pinching was performed to ensure animals did not respond before proceeding with exsanguination, which was performed through blood collection from the left ventricle. The vasculature of the animals was then perfused with cold sterile PBS (without calcium or magnesium) to ensure all blood was removed from the organs.

The lungs were collected for several endpoints, including metals and immunofluorescence analyses. Specifically, the upper left lung lobe was dissected for metals analyses. The lobe was rinsed with PBS to remove any residual blood and blotted dry. A section was further dissected, weighed and placed in a trace element-free tube at −80 °C until it was digested for metals analyses. We previously reported the metal levels from this model [36].

Seven male and seven female guinea pigs were considered for histological and immunofluorescence endpoints. The right lung was inflated and fixed with 10% neutral buffered formalin using a gravity feeder. This method ensures the preservation of the lung anatomy. Briefly, a suture was tied around the left bronchus. A cannula was inserted in the trachea and secured with a suture. The cannula was connected with a tube and a syringe was placed at about a 25 cm height. Slowly, the clamp was removed from the tubing, allowing the 10% neutral buffered formalin to enter the lungs until the pressures reached an equilibrium and were left at this state for 10 min. The clamp was placed on the tubing and the suture around the trachea was tightened while removing the cannula. The next day, the lungs were washed 4 times with 1× PBS to remove any remaining blood and immediately placed in a container with 70% ethanol until they were ready to be further dissected, paraffin-embedded and sectioned.

### 4.5. Immunofluorescence Endpoints for DNA Double-Strand Breaks and Repair

Formalin-fixed, paraffin-embedded lungs were sectioned and stained to visualize DNA double-strand breaks and repair markers as previously described, with some modifications [11]. DNA double-strand breaks were measured using gamma-H2AX (γH2AX) [20,21,22]. DNA repair was analyzed by quantifying foci levels of RAD51, which is a key protein in homologous recombination repair that binds to single-stranded DNA and forms nuclear filaments that guide the strand exchange and homology search [23]. RAD51 foci are considered a functional marker for homologous recombination repair [65,66]. RAD51 is known to be inhibited by Cr(VI) exposure and thus is a key step in the carcinogenic mechanism of Cr(VI). In addition, cytokeratin was used as an epithelial cell marker.

For the immunofluorescence staining, each staining batch included one male and one female from each exposure group (n = 8 total) and one slide was also added per batch as a negative control. The latter was only stained with secondary antibodies. Control values are comparable across batches, as they are reflected by low SEM values in the controls, indicating low variability between batches. Thus, exposure group and sex were not confounded with the staining batch.

All slides were baked for 20 min at 60 °C, deparaffinized using xylene and rehydrated using a gradient of ethanol that ended with water. Tissue slides were then incubated in a steamer for 30 min in an antigen retrieval solution that consisted of 10 mM Tris-1 mM EDTA (pH 9) with 0.05% Tween-20. After the incubation, the slides were allowed to cool down at room temperature for 45 min and washed 3 times for 5 min each using Tris Buffered Saline (TBS) (Sigma-Aldrich, St. Louis, MI, USA) with 0.025% Triton X-100 (TBS-T). After the washes, staining protocols for γH2AX and RAD51 slightly differed.

For γH2AX, slides were blocked using a TrueBlack Blocking Buffer (Biotium, Fremont. CA. USA, 23012) for 15 min at room temperature. Slides were then incubated overnight with the primary antibody cocktail consisting of γH2AX (rabbit, 1:100, Cell Signaling, Danvers, MA, USA, 2577S) and cytokeratin (mouse, 1:100, Sigma-Aldrich, St. Louis, MI, USA, C2931) antibodies in a TrueBlack Background Suppressor Buffer (Biotium, Fremont, CA, USA). After overnight incubation, slides were washed for 5 min, three times, using TBS-T. Slides were incubated for 1 h in the secondary antibody cocktail consisting of goat anti-rabbit Alexa Fluor 488 (1:500, Life Technologies, Carlsbad, CA, USA, A11034) and goat anti-mouse Alexa Fluor 555 (1:500, Life Technologies, Carlsbad, CA, USA, A21422). Then, slides were washed 3 times with TBS and incubated in 1% Sudan Black B in 70% ethanol for 10 min. Slides were then rinsed with TBS-T, washed for 5 min in TBS-T and rinsed several times in TBS. Then, slides were incubated with 10 μg/mL DAPI (Biolegend, San Diego, CA, USA, 422801) in TBS for 30 min before being rinsed with TBS twice and mounted with No 1.5 coverslips using ProLong^TM^ Diamond Antifade Mountant (Invitrogen, Carlsbad, CA, USA, P36971) with DAPI. The slides were then allowed to cure overnight and nail polish was applied to the sides of the coverslip. Slides were stored at 4 °C until imaging.

For RAD51, slides were incubated with Image-iT^TM^ FX Signal Enhancer (Thermo Fisher, Waltham, MA, USA) for 30 min and washed with TBS twice for 5 min each. Slides were then blocked with TrueBlack Blocking Buffer for 15 min at room temperature and incubated with the primary antibodies for 2 h. The antibody cocktail consisted of RAD51 (rabbit, 1:10,000, Thermo Fisher, Waltham, MA, USA, PA5-27195) and cytokeratin (mouse, 1:100) antibodies in TrueBlack Background Suppressor Buffer. After incubation, the slides were washed three times with TBST for 5 min each. Then, the slides were incubated for 1 h at room temperature with secondary antibodies, including goat anti-rabbit Alexa Fluor 488 (1:500) and goat anti-mouse Alexa Fluor 555 (1:500) diluted in TrueBlack Background Suppressor Buffer. Next, slides were washed with TBS 3 times for 5 min each and placed in 1% Sudan Black B solution in 70% ethanol for 10 min. After the incubation, slides were rinsed with TBS-T, washed for 5 min in TBS-T and rinsed again with TBS before they were incubated again with 10 μg/mL DAPI in TBS for 30 min. Then, the slides were rinsed in TBS twice and mounted with No 1.5 coverslips using ProLong^TM^ Diamond Antifade Mountant with DAPI. The slides were allowed to cure overnight and nail polish was applied to the sides of the coverslip. Slides were stored at 4 °C until imaging.

### 4.6. Confocal Imaging and Foci Analyses

Two regions of the lungs were considered: the bronchioles and the alveoli. One slide containing one formalin-fixed paraffin-embedded section (5 µm thickness) per animal was considered per endpoint. These lung regions were imaged using a Nikon A1 Confocal Microscope Ti with the NIS Elements software (ver. AR 5.20.02). The bronchioles were defined as the airways in between the bronchi and the terminal respiratory bronchioles, where bronchi are characterized by having extensive cartilage plaques and respiratory bronchioles are closest to the alveoli. Stitched images of the bronchioles were obtained at 100× at a single focal plane or depth of the tissue; a grid was placed over and sequential images were obtained along the bronchioles using 100× with a 3× digital zoom. The alveoli surrounding the bronchioles in each animal were also imaged. Because the image was obtained at a single plane of depth, the person acquiring the image could not visualize the foci within the tissue. In addition, the LUTs were adjusted for the target marker so that they were not visible during field selection, while DAPI and the cellular markers were. Adjusting the LUTs changes how an image looks on the monitor without altering the raw data collected by the confocal. Images were acquired consecutively along the bronchiolar epithelium and alveoli, with no gaps within the adjacent fields; only a small margin was allowed between images to prevent the same cells from being imaged twice. Images of bronchioles and alveoli were taken with a 100× objective and a 3× digital zoom. Z-stack images were obtained with a 1 µm step size for γH2AX and 0.75 µm for RAD51. The same laser settings were used across slides within a staining batch. The batch information is described above. Only images containing whole nuclei were considered for analysis. The z-stack images obtained at 100× with digital zoom were then denoised (Denoise AI tool) and blinded by another lab member prior to scoring. The foci were manually scored one cell at a time, layer by layer in the z-stack. The scorer had no information regarding the exposure group or sex. The Denoise AI tool reduces the background noise of images without altering the raw data. Nuclear foci were scored in 100 nuclei per region in each animal using the z-stack images. Foci defined as distinct, clearly identifiable dots of signal above background in the nuclear region were counted as foci. The foci data are represented as total foci in 100 nuclei.

### 4.7. Statistical Analysis

For comparisons of one variable (e.g., dose of zinc chromate), the distribution of the data was assessed. For data with a normal distribution, the Student’s *t*-test with Welch’s correction (parametric test) was used, while for non-normally distributed data, the Mann–Whitney U test (non-parametric test) was used. Statistical significance (*p*) was reported for values below 0.05; however, statistical results for values below 0.1 are also provided for comparison, as they are close to achieving significance. For the statistical analyses considering sex differences, a two-way ANOVA was performed, followed by a multiple comparison test. Specifically, to correct for multiple comparisons by controlling the False Discovery Rate (FDR) the two-stage step-up method of Benjamini, Krieger and Yekutieli was used. These results are reported as *q*-values, with significance established at those below 0.05, but 0.1 is also provided for comparison. In the figures below, graphs A and B provide *p* values and C and D graphs provide *q* values. All graphs and statistical analyses were performed using the GraphPad Prism 11.0.2 software. Data were reported as mean ± standard error of the mean (SEM). SEM was used to represent the precision of the estimated group average rather than the variability. However, the individual data points are also presented in each graph to show the spread of the data.

To directly analyze the outcomes observed for DNA double-strand breaks and DNA repair in guinea pigs after acute zinc chromate exposure, we used a DNA double-strand break repair ratio. For this ratio, the levels of DNA double-strand breaks (total γH2AX foci) in an animal are divided by the levels of repair (total RAD51 foci) in the same animal. A decrease in this ratio shows an increase in DNA repair, indicated by a decrease in DNA breaks or an increase in RAD51 levels. On the contrary, an increase in the ratio indicates an inhibition of repair, as the number of DNA breaks increases relative to RAD51 levels.

## 5. Conclusions

Cr(VI) is a known human lung carcinogen with widespread exposure risks. Despite it being a human health hazard, how it causes lung cancer is unclear. Our study has, for the first time, translated the mechanism of Cr(VI)-induced DNA double-strand breaks and inhibition of homologous recombination repair to guinea pig lungs after subchronic exposure to particulate Cr(VI) via oropharyngeal aspiration. Specifically, we have shown that the mechanism translates to bronchiolar epithelial cells and not to alveolar cells, which is consistent with Cr(VI) causing tumors at the bronchial bifurcation where particles impact. Future studies will consider how Cr(VI) inhibits RAD51 and how this inhibition affects genomic stability.

## Figures and Tables

**Figure 1:**
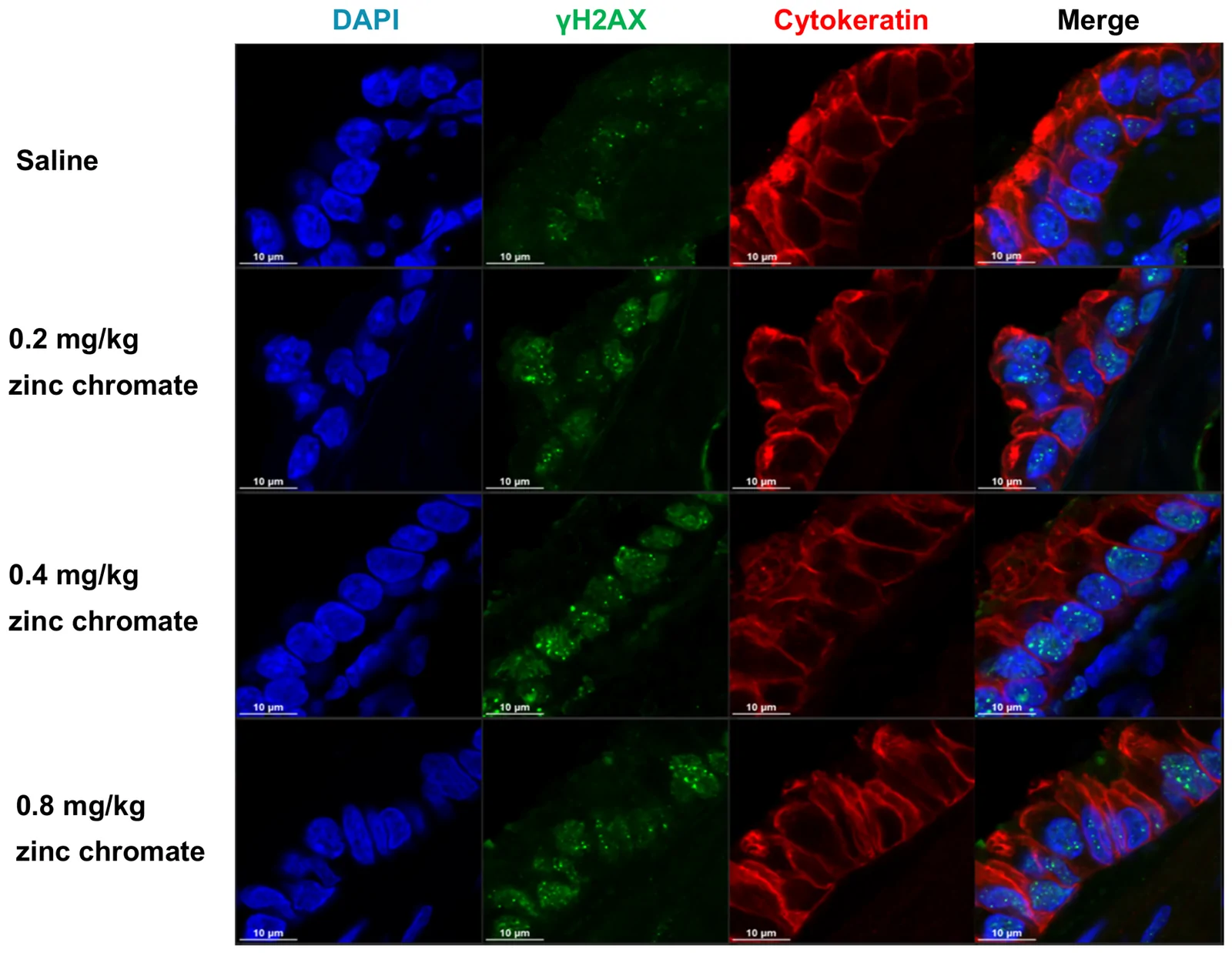
Acute exposure to zinc chromate induces γH2AX foci in the bronchioles of guinea pigs. The figure shows representative images of γH2AX foci in the bronchioles of guinea pigs acutely exposed to saline or various zinc chromate concentrations. Images were obtained using a confocal microscope with a ×100 objective and a ×3 digital zoom. The images represent one z-stack layer. Scale bars represent 10 μm. The targets are organized by column: the first column (blue) is DAPI treatment to stain the nucleus; the second column (green) is γH2AX foci, a marker of DNA double-strand breaks; the third column (red) is cytokeratin expression, a marker of epithelial cells; and the fourth column is the merged image. The images were cropped and rearranged to display the merged image last.

**Figure 2:**
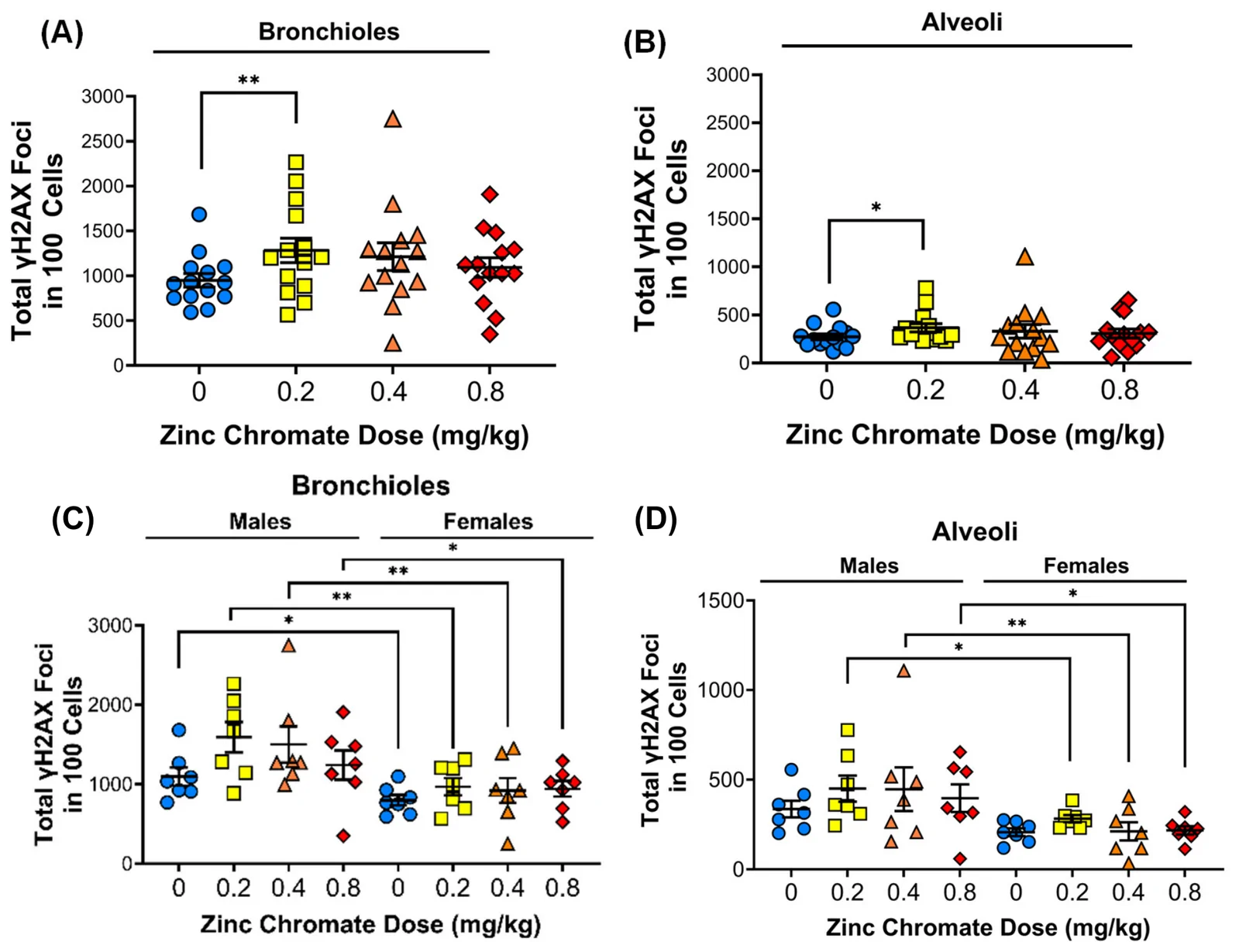
Acute exposure to zinc chromate induces DNA double-strand breaks in lung epithelial cells. The data in these graphs show DNA double-strand break levels in bronchiolar and alveolar epithelial cells of guinea pigs acutely exposed to zinc chromate. The horizontal lines in the graphs represent the mean values and each error bar is the standard error of the mean. Each dot represents one animal, and 7 males and 7 females were analyzed per exposure group. Symbols represent different exposure groups: saline (blue circles), 0.2 mg/kg zinc chromate (yellow squares), 0.4 mg/kg zinc chromate (orange triangles) and 0.8 mg/kg zinc chromate (red diamonds). Asterisks (*) show statistical results, where * *p* or *q* ≤ 0.1, and ** *p* or *q* < 0.05. All graphs show the total γH2AX foci levels in 100 cells. (**A**) γH2AX in bronchioles. (**B**) γH2AX in alveoli. (**C**) γH2AX in bronchioles of male and female guinea pigs, separately. (**D**) γH2AX in alveoli of male and female guinea pigs, separately.

**Figure 3:**
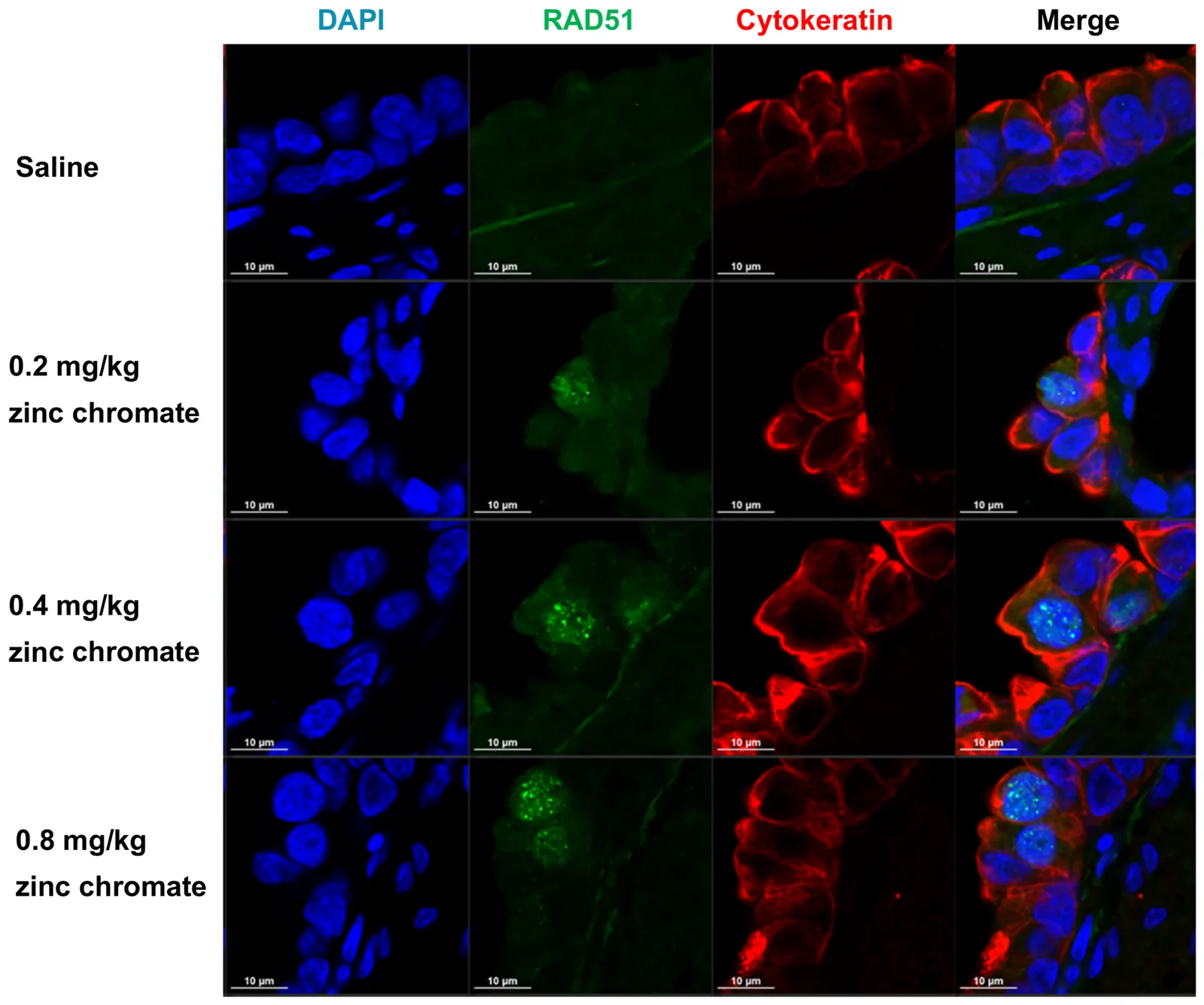
Acute exposure to zinc chromate induces RAD51 foci in the bronchioles of guinea pigs. The figure shows representative images of RAD51 foci in the bronchioles of guinea pigs acutely exposed to saline or various zinc chromate concentrations. Images were obtained using a confocal microscope with a ×100 objective and a ×3 digital zoom. The images represent one z-stack layer. Scale bars represent 10 μm. The targets are organized by column: the first column (blue) is DAPI treatment to stain the nucleus; the second column (green) is RAD51 foci, a marker of homologous recombination repair; the third column (red) is cytokeratin expression, a marker of epithelial cells; and the fourth column is the merged image. The images were cropped and rearranged to display the merged image last.

**Figure 4:**
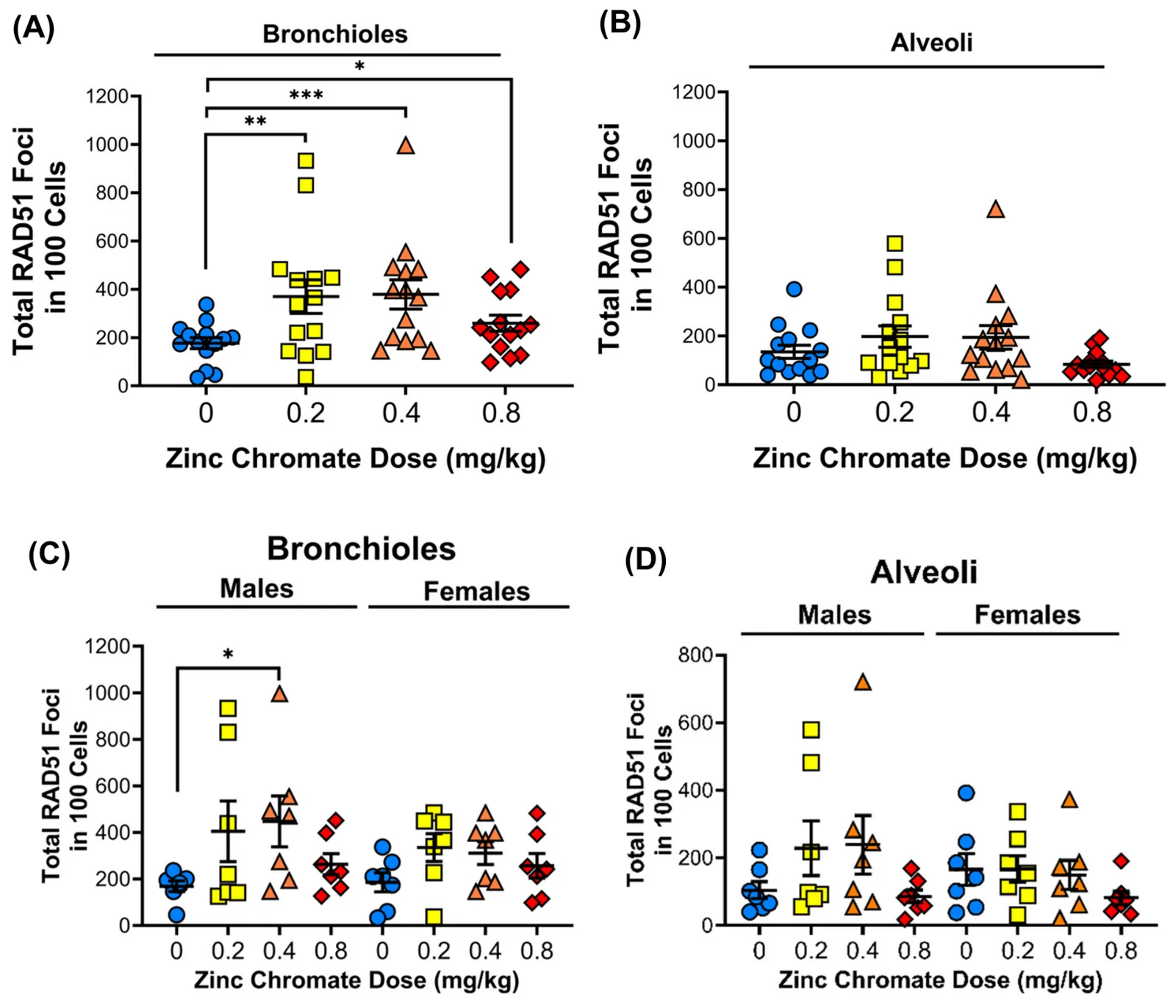
Acute exposure to zinc chromate induces homologous recombination repair in the bronchioles but not in the alveoli. The data in these graphs show RAD51 foci levels, indicative of DNA repair, in the bronchioles and alveoli of guinea pigs acutely exposed to zinc chromate. The horizontal lines in the graphs represent the mean values and each error bar is the standard error of the mean. Each dot represents one animal, and 7 males and 7 females were analyzed per exposure group. Symbols represent different exposure groups: saline (blue circles), 0.2 mg/kg zinc chromate (yellow squares), 0.4 mg/kg zinc chromate (orange triangles) and 0.8 mg/kg zinc chromate (red diamonds). Asterisks (*) show statistical results, where * *p* or *q* ≤ 0.1, ** *p* or *q* < 0.05, and *** *p* or *q* < 0.01. All graphs show the total RAD51 foci levels in 100 cells. (**A**) RAD51 in bronchioles. (**B**) RAD51 in alveoli. (**C**) RAD51 in bronchioles of male and female guinea pigs, separately. (**D**) RAD51 in alveoli of male and female guinea pigs, separately.

**Figure 5:**
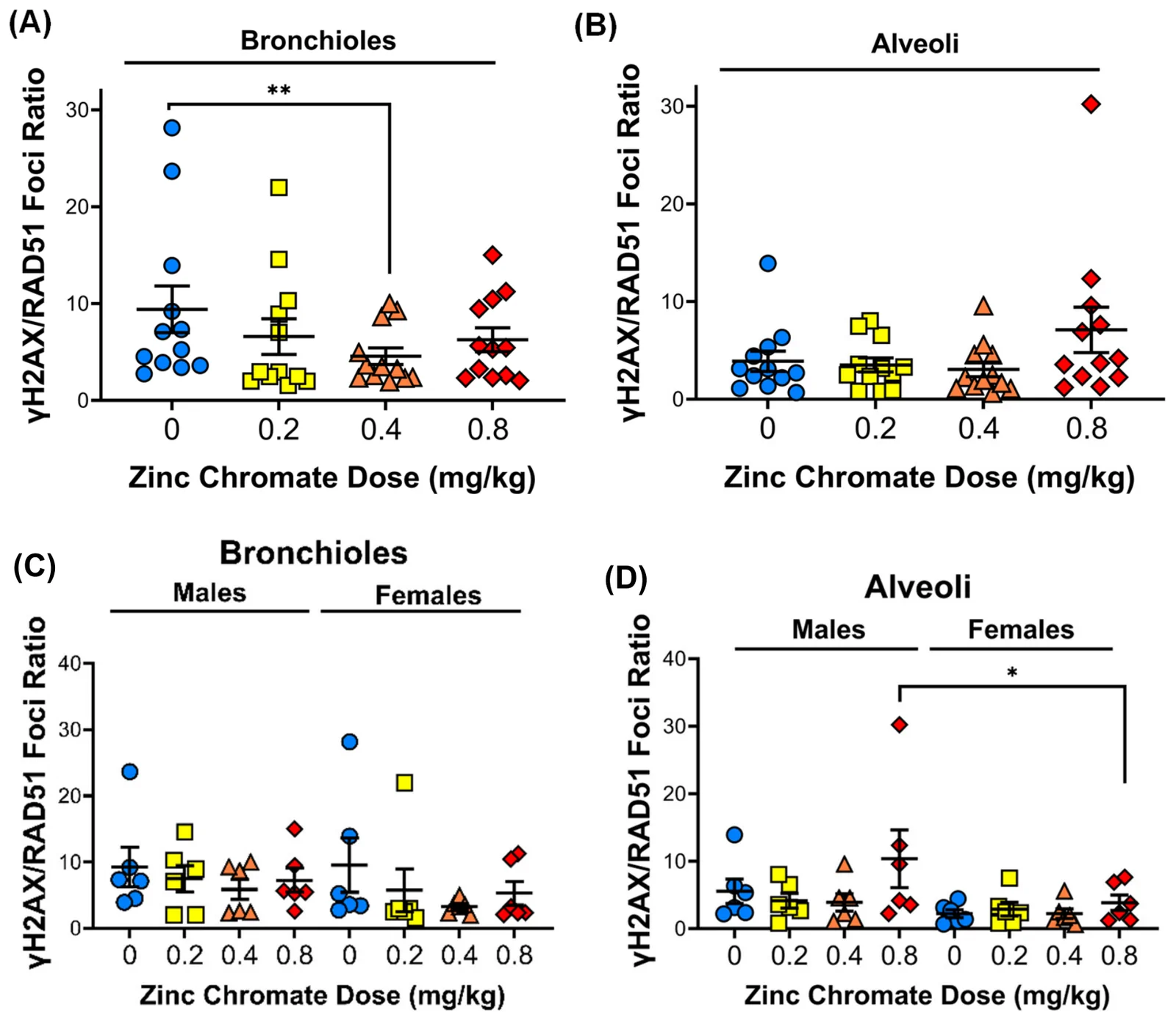
Acute exposure to zinc chromate induces DNA repair in the bronchioles. The data in these graphs show the DNA repair ratio, calculated by dividing the levels of γH2AX by RAD51 foci levels. A decrease in this ratio indicates repair, while an increase indicates inhibition of repair. The horizontal lines in the graphs represent the mean values and each error bar is the standard error of the mean. Each dot represents one animal, and 6 males and 6 females were analyzed per exposure group. Symbols represent different exposure groups: saline (blue circles), 0.2 mg/kg zinc chromate (yellow squares), 0.4 mg/kg zinc chromate (orange triangles) and 0.8 mg/kg zinc chromate (red diamonds). Asterisks (*) show statistical results, where * *p* or *q* ≤ 0.1, and ** *p* or *q* < 0.05. All graphs show the γH2AX/RAD51 repair ratio. (**A**) DNA repair ratio in bronchioles. (**B**) DNA repair ratio in alveoli. (**C**) DNA repair ratio in bronchioles of male and female guinea pigs, separately. (**D**) DNA repair ratio in alveoli of male and female guinea pigs, separately.

**Figure 6:**
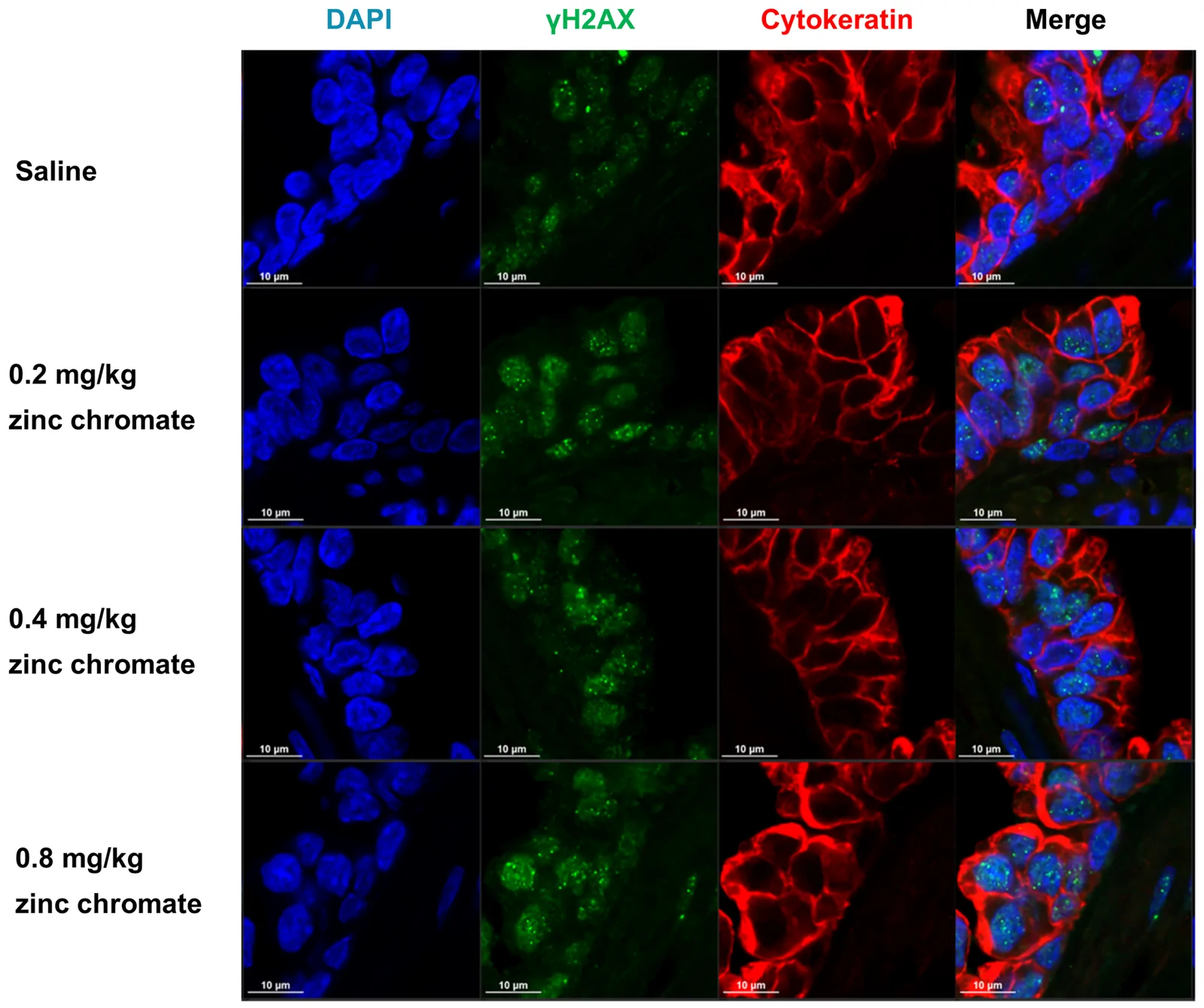
Subchronic exposure to zinc chromate increases the levels of DNA double-strand breaks in the bronchioles of guinea pigs. The figure shows representative images of γH2AX foci, indicative of DNA double-strand breaks, in the bronchioles of guinea pigs subchronically exposed to saline or various zinc chromate concentrations. Images were obtained using a confocal microscope with an ×100 objective and an ×3 digital zoom. The images represent one z-stack layer. Scale bars represent 10 μm. The targets are organized by column: the first column (blue) is DAPI treatment to stain the nucleus; the second column (green) is γH2AX foci, a marker of DNA double-strand breaks; the third column (red) is expression of cytokeratin, a marker of epithelial cells; and the fourth column is the merged image. The images were cropped and rearranged to display the merged image last.

**Figure 7:**
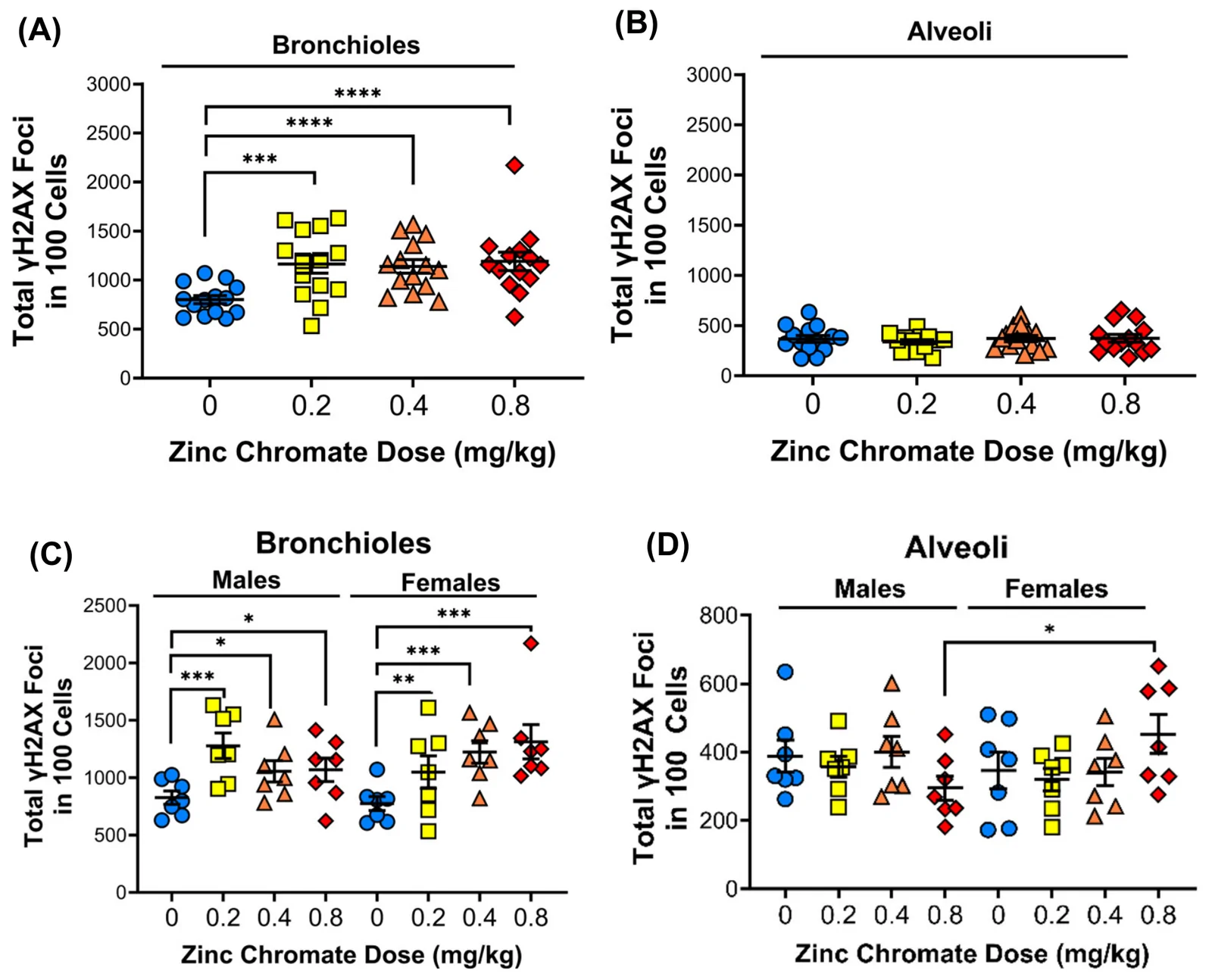
Subchronic exposure to zinc chromate induces DNA double-strand break levels in the bronchioles. The data in these graphs show DNA double-strand break levels in bronchiolar and alveolar epithelial cells of guinea pigs subchronically exposed to zinc chromate. The horizontal lines in the graphs represent the mean values and each error bar is the standard error of the mean. Each dot represents one animal, and 7 males and 7 females were analyzed per exposure group. Symbols represent different exposure groups: saline (blue circles), 0.2 mg/kg zinc chromate (yellow squares), 0.4 mg/kg zinc chromate (orange triangles) and 0.8 mg/kg zinc chromate (red diamonds). Asterisks (*) show statistical results, where * *p* or *q* ≤ 0.1, ** *p* or *q* < 0.05, *** *p* or *q* < 0.01, and **** *p* or *q* < 0.001. All graphs show the total γH2AX foci levels in 100 cells. (**A**) γH2AX in bronchioles. (**B**) γH2AX in alveoli. (**C**) γH2AX in bronchioles of male and female guinea pigs, separately. (**D**) γH2AX in alveoli of male and female guinea pigs, separately.

**Figure 8:**
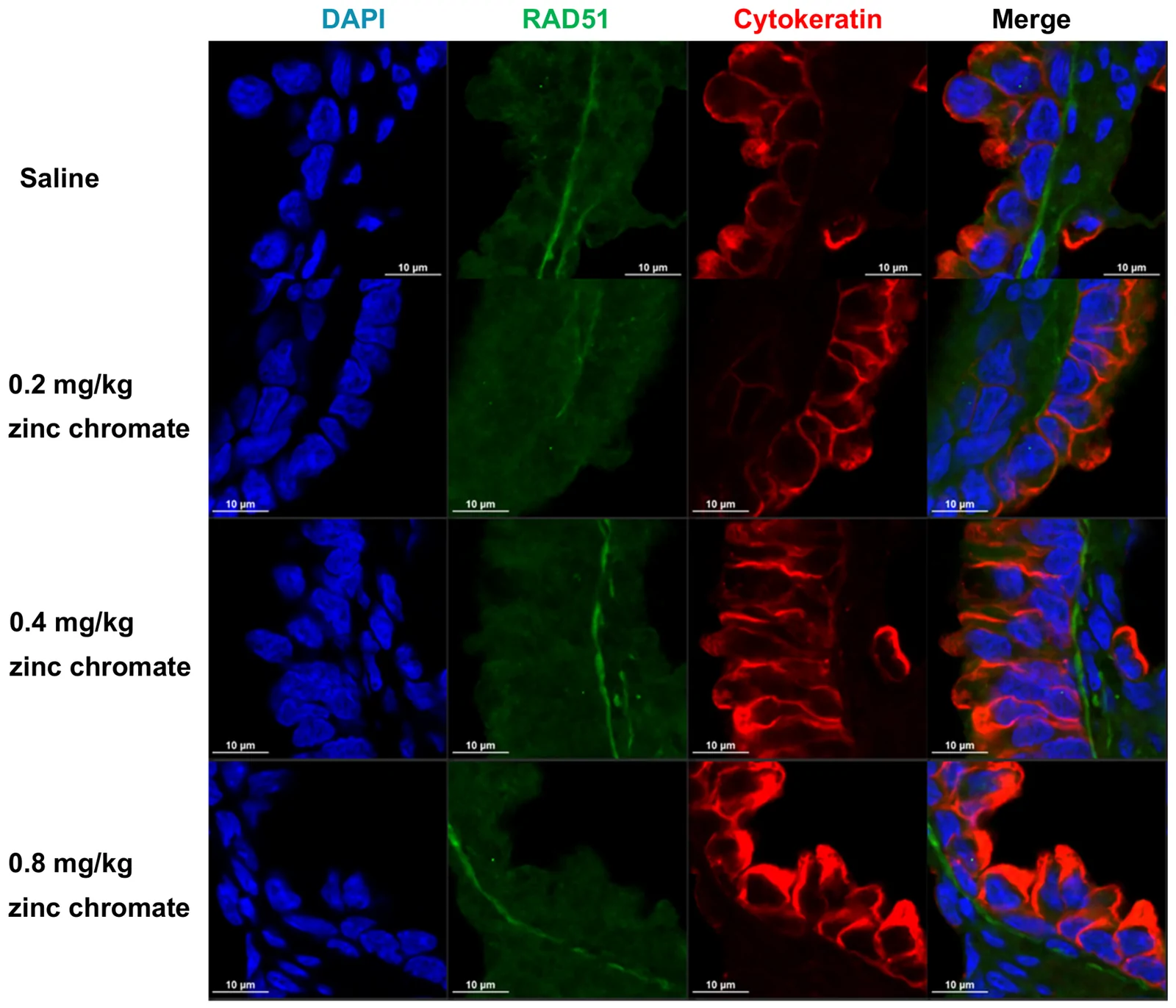
Subchronic exposure to zinc chromate inhibits RAD51 foci in the bronchioles of guinea pigs. The figure shows representative images of RAD51 foci in the bronchioles of guinea pigs subchronically exposed to saline or various zinc chromate concentrations. Images were obtained using a confocal microscope with an ×100 objective and an ×3 digital zoom. The images represent one z-stack layer. Scale bars represent 10 μm. The targets are organized by column: the first column (blue) is DAPI treatment to stain the nucleus; the second column (green) is RAD51 foci, a marker of homologous recombination repair; the third column (red) is expression of cytokeratin, a marker of epithelial cells; and the fourth column is the merged image. The images were cropped and rearranged to display the merged image last.

**Figure 9:**
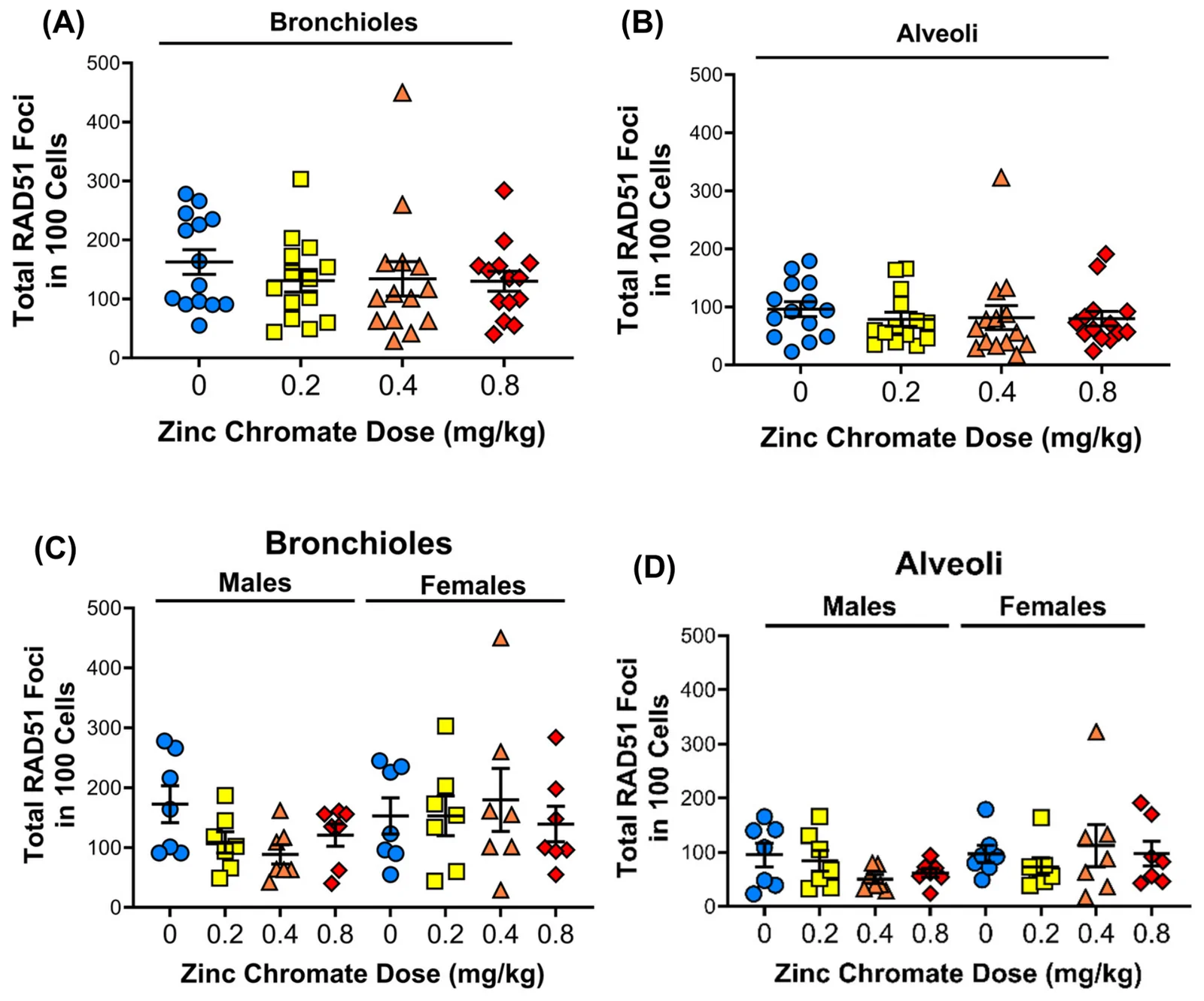
Subchronic exposure to zinc chromate inhibits homologous recombination repair. The data in these graphs show RAD51 foci levels in the bronchioles and alveoli of guinea pigs subchronically exposed to zinc chromate. The horizontal lines in the graphs represent the mean values and each error bar is the standard error of the mean. Each dot represents one animal, and 7 males and 7 females were analyzed per exposure group. Symbols represent different exposure groups: saline (blue circles), 0.2 mg/kg zinc chromate (yellow squares), 0.4 mg/kg zinc chromate (orange triangles) and 0.8 mg/kg zinc chromate (red diamonds). All graphs show the total RAD51 foci levels in 100 cells. (**A**) RAD51 in bronchioles. (**B**) RAD51 in alveoli. (**C**) RAD51 in bronchioles of male and female guinea pigs, separately. (**D**) RAD51 in alveoli of male and female guinea pigs, separately.

**Figure 10:**
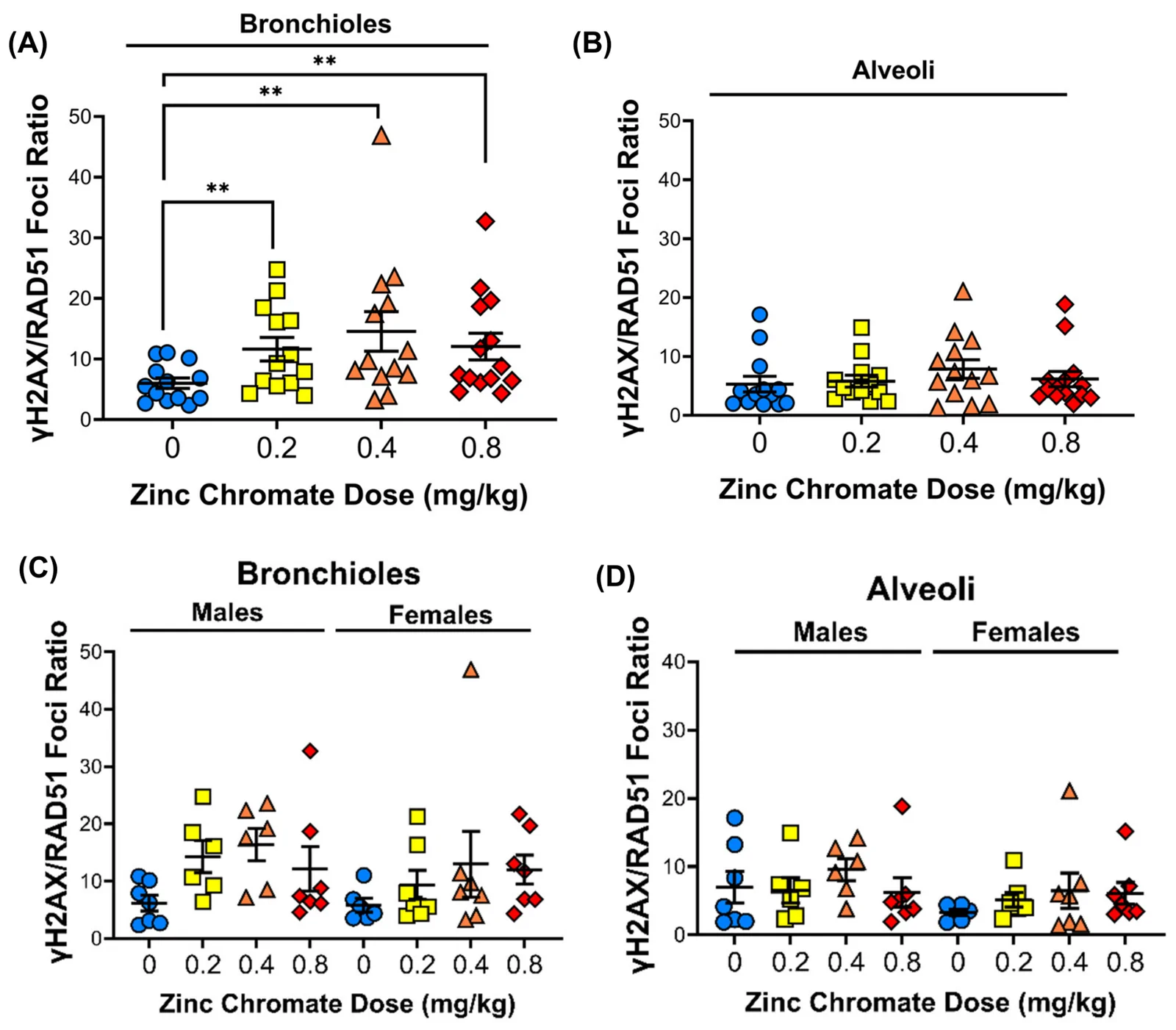
Subchronic exposure to zinc chromate inhibits DNA repair in the bronchioles. The data in these graphs show the DNA repair ratio, calculated by dividing the levels of γH2AX by RAD51 foci levels. A decrease in this ratio indicates repair, while an increase indicates inhibition of repair. The horizontal lines in the graphs represent the mean values and each error bar is the standard error of the mean. Each dot represents one animal, and 6 to 7 males and 6 to 7 females were analyzed per exposure group. Symbols represent different exposure groups: saline (blue circles), 0.2 mg/kg zinc chromate (yellow squares), 0.4 mg/kg zinc chromate (orange triangles) and 0.8 mg/kg zinc chromate (red diamonds). Asterisks (*) show statistical results, where ** *p* or *q* < 0.05. All graphs show the γH2AX/RAD51 repair ratio. (**A**) DNA repair ratio in bronchioles. (**B**) DNA repair ratio in alveoli. (**C**) DNA repair ratio in bronchioles of male and female guinea pigs, separately. (**D**) DNA repair ratio in alveoli of male and female guinea pigs, separately.

**Figure 11:**
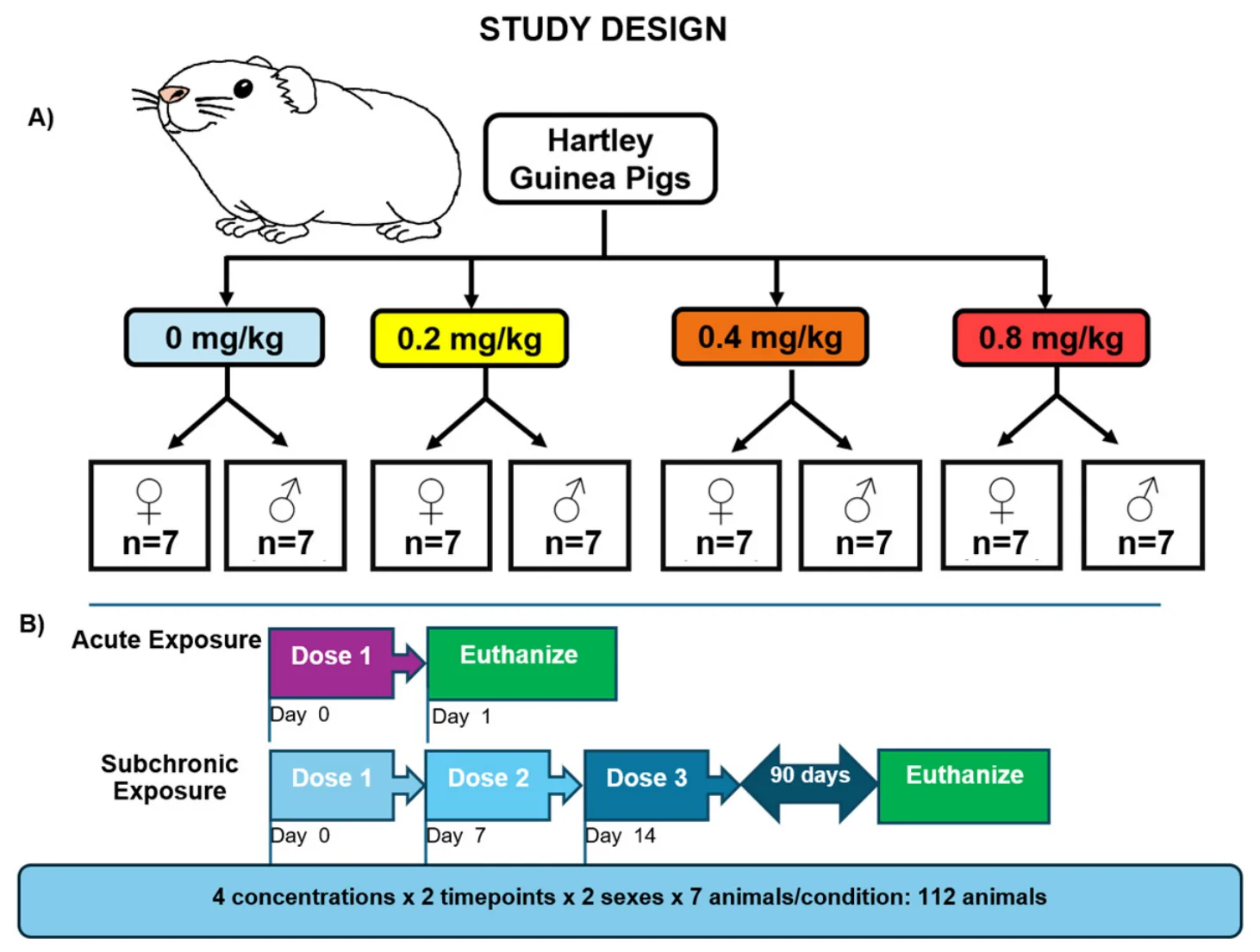
Schematic of the study design. (**A**) Treatments and number of animals per group. (**B**) Exposure design and duration.

## Data Availability

All the original contributions presented in this study are included in the article and further inquiries can be directed to the corresponding author.

## Supplementary Materials

The following supporting information can be downloaded at: https://www.mdpi.com/article/10.3390/ijms27198529/s1.

## Abbreviations

The following abbreviations are used in this manuscript:

DNA	Deoxyribonucleic acid
RAD51	DNA repair protein RAD51 homolog 1
Cr(VI)	Hexavalent chromium
Cr(III)	Trivalent chromium
γH2AX	Gamma-H2A.X variant histone
DAPI	4′,6-diamidino-2-phenylindole
EDTA	Ethylenediaminetetraacetic acid
TBS	Tris buffered saline
