# Supplementary material for: Mechanisms of Hexavalent Chromium-Induced Lung Carcinogenesis: DNA Double-Strand Breaks and DNA Repair Inhibition in a Guinea Pig Model
Source: Int J Mol Sci. 2026 Sep 24;27(19):8529. doi: 10.3390/ijms27198529 (PMC13664759; doi:10.3390/ijms27198529)
Supplement: Supplementary file 1 [file ijms-27-08529-s001.zip › ijms-4500583-supplementary.pdf]

Supplementary materials:

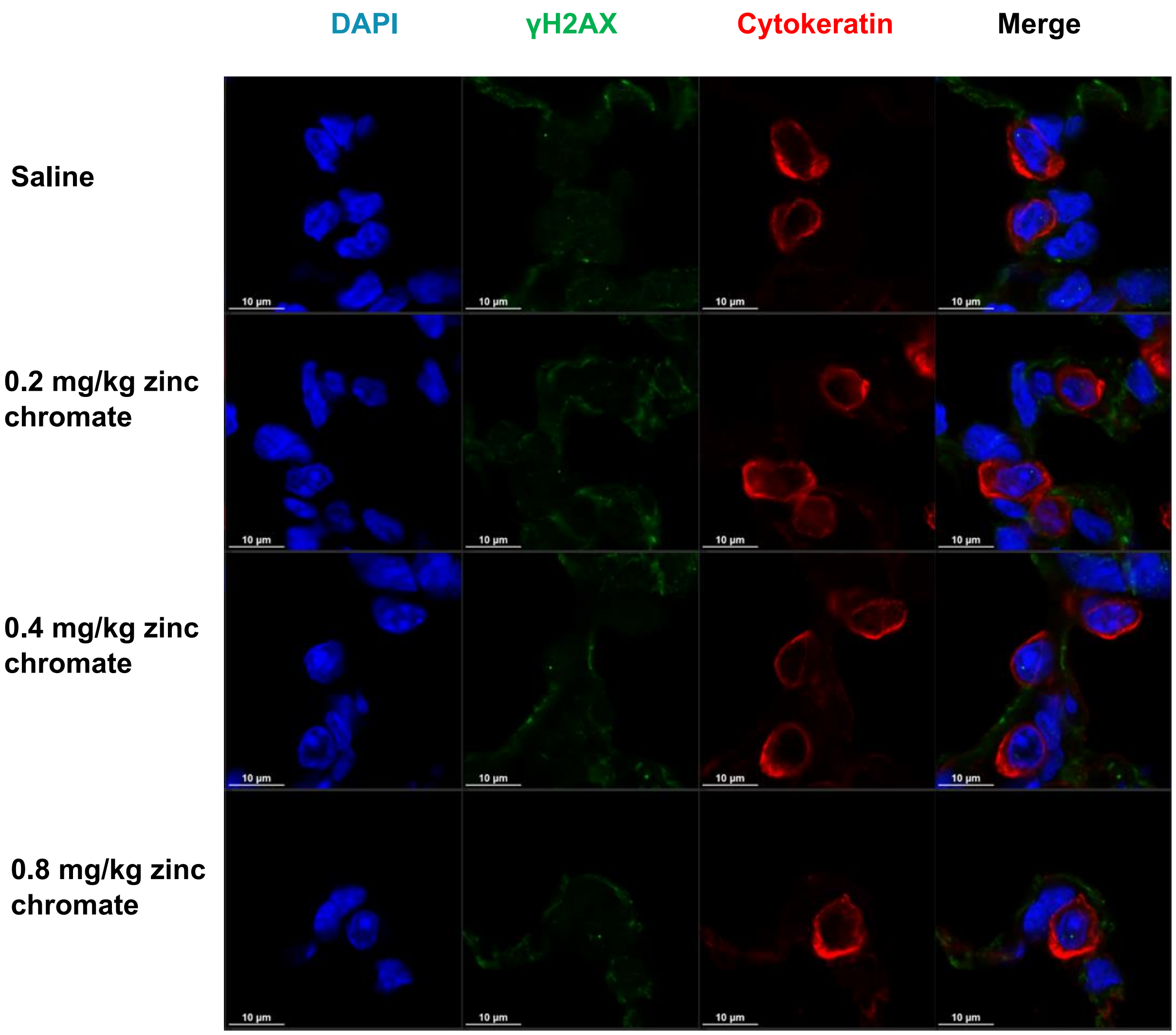

Figure S1: Representative images of γH2AX after acute exposure in the alveoli.

The figure shows representative images of DNA double-strand breaks measured by γH2AX foci quantification in the alveoli of guinea pigs acutely exposed to saline or various zinc chromate concentrations. Images were obtained using a confocal microscope with an x100 objective and an x3 digital zoom. Scale bars represent 10 um. The targets are organized by column: the first column is DAPI treatment to stain the nucleus; the second column is γH2AX foci, a marker of DNA double strand breaks; the third column is cytokeratin expression, a marker of epithelial cells; the fourth column is the merged image. The images were cropped and rearranged to display the merged image last.

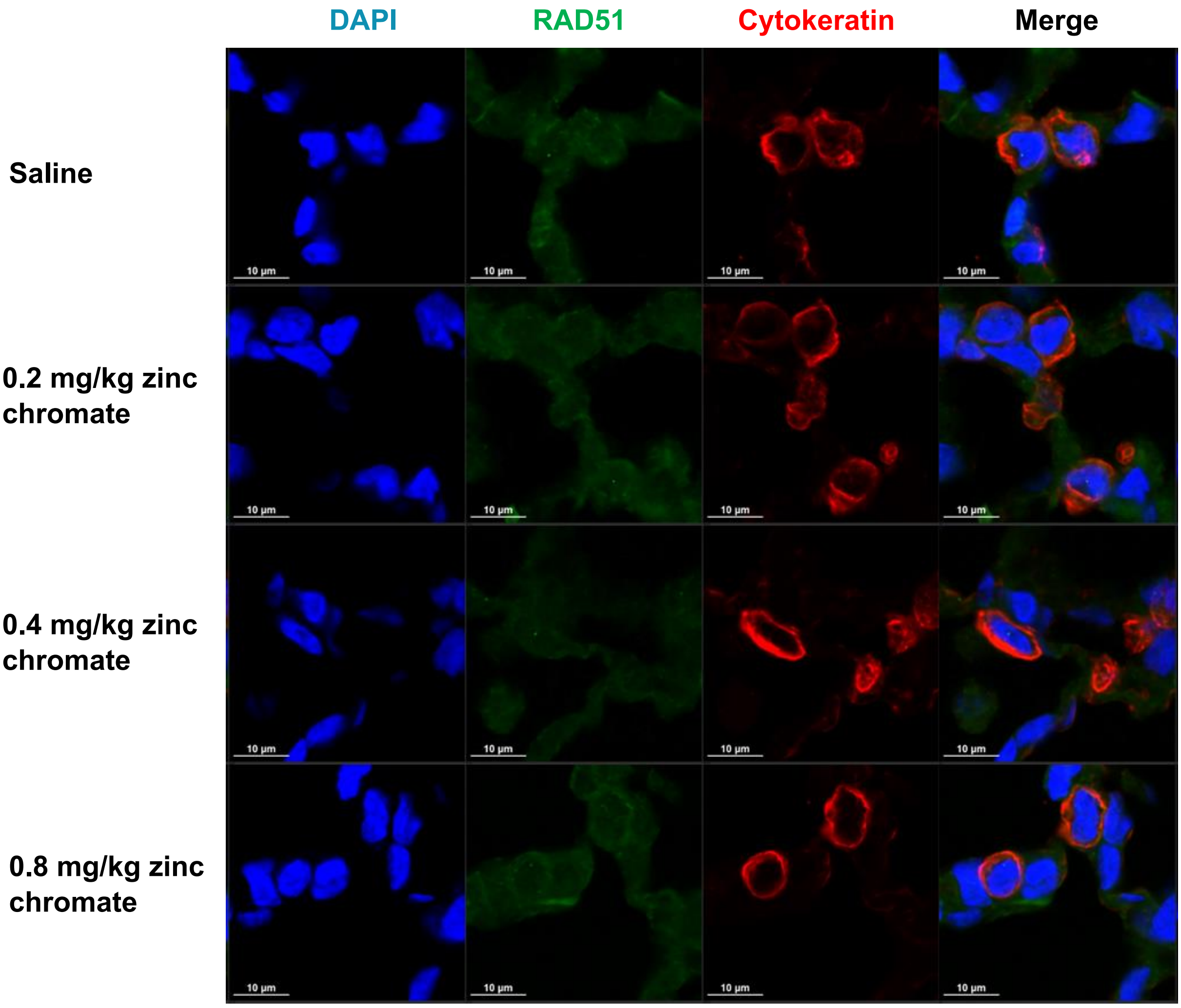

Figure S2. Representative images of RAD51 after acute exposure in the alveoli.

The figure shows representative images of RAD51 foci in the alveoli of guinea pigs acutely exposed to saline or various zinc chromate concentrations. Images were obtained using a confocal microscope with a x100 objective and a x3 digital zoom. Scale bars represent 10 um. The targets are organized by column: the first column is DAPI treatment to stain the nucleus; the second column is RAD51 foci, a marker of homologous recombination repair; the third column is cytokeratin expression, a marker of epithelial cells; the fourth column is the merged image. The images were cropped and rearranged to display the merged image last.

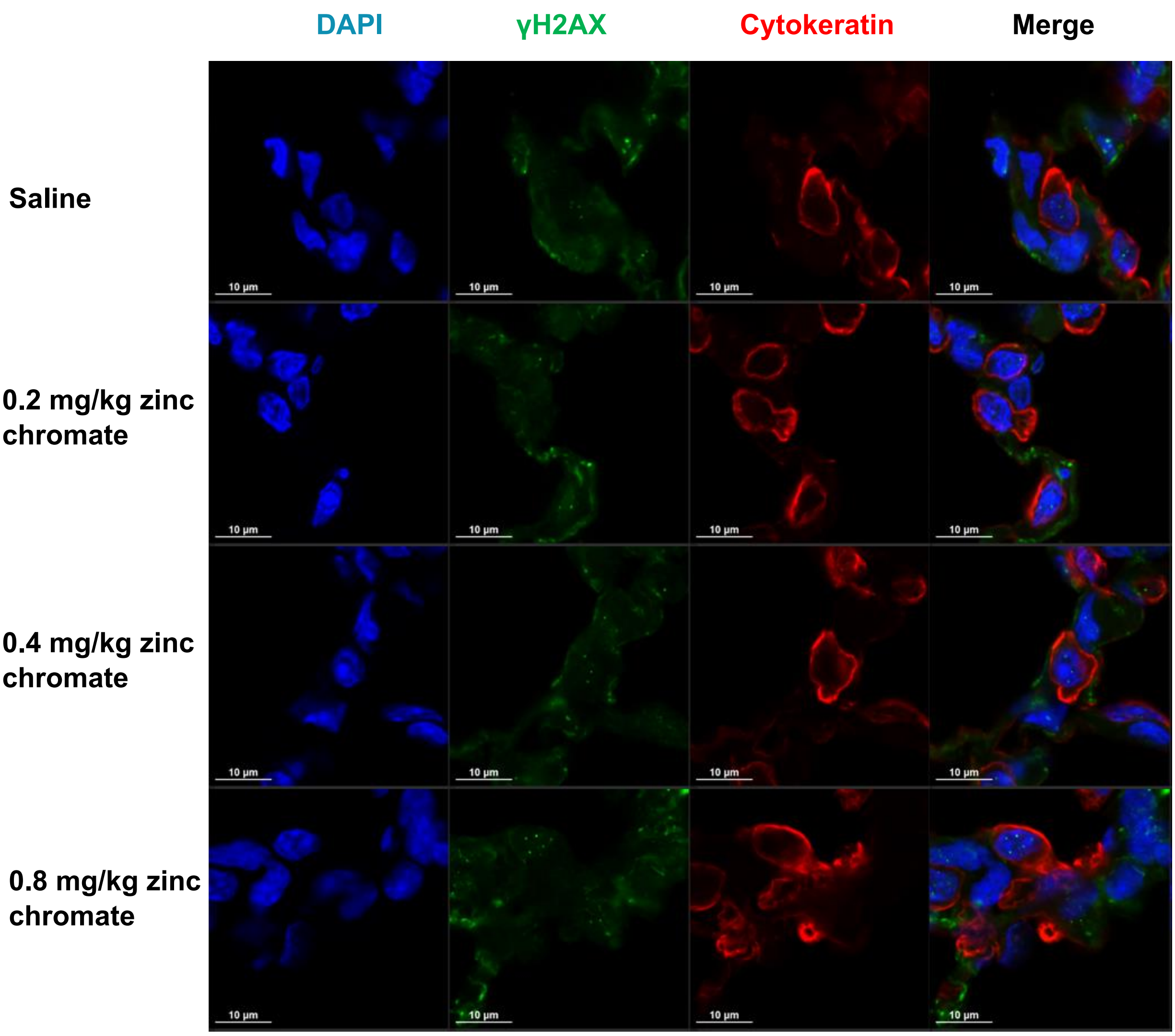

Figure S3: Representative images of γH2AX after subchronic exposure in the alveoli.

The figure shows representative images of DNA double-strand breaks measured by γH2AX foci quantification in the alveoli of guinea pigs subchronically exposed to saline or various zinc chromate concentrations. Images were obtained using a confocal microscope with an x100 objective and an x3 digital zoom. Scale bars represent 10 um. The targets are organized by column: the first column is DAPI treatment to stain the nucleus; the second column is γH2AX foci, a marker of DNA double strand breaks; the third column is cytokeratin expression, a marker of epithelial cells; the fourth column is the merged image. The images were cropped and rearranged to display the merged image last.

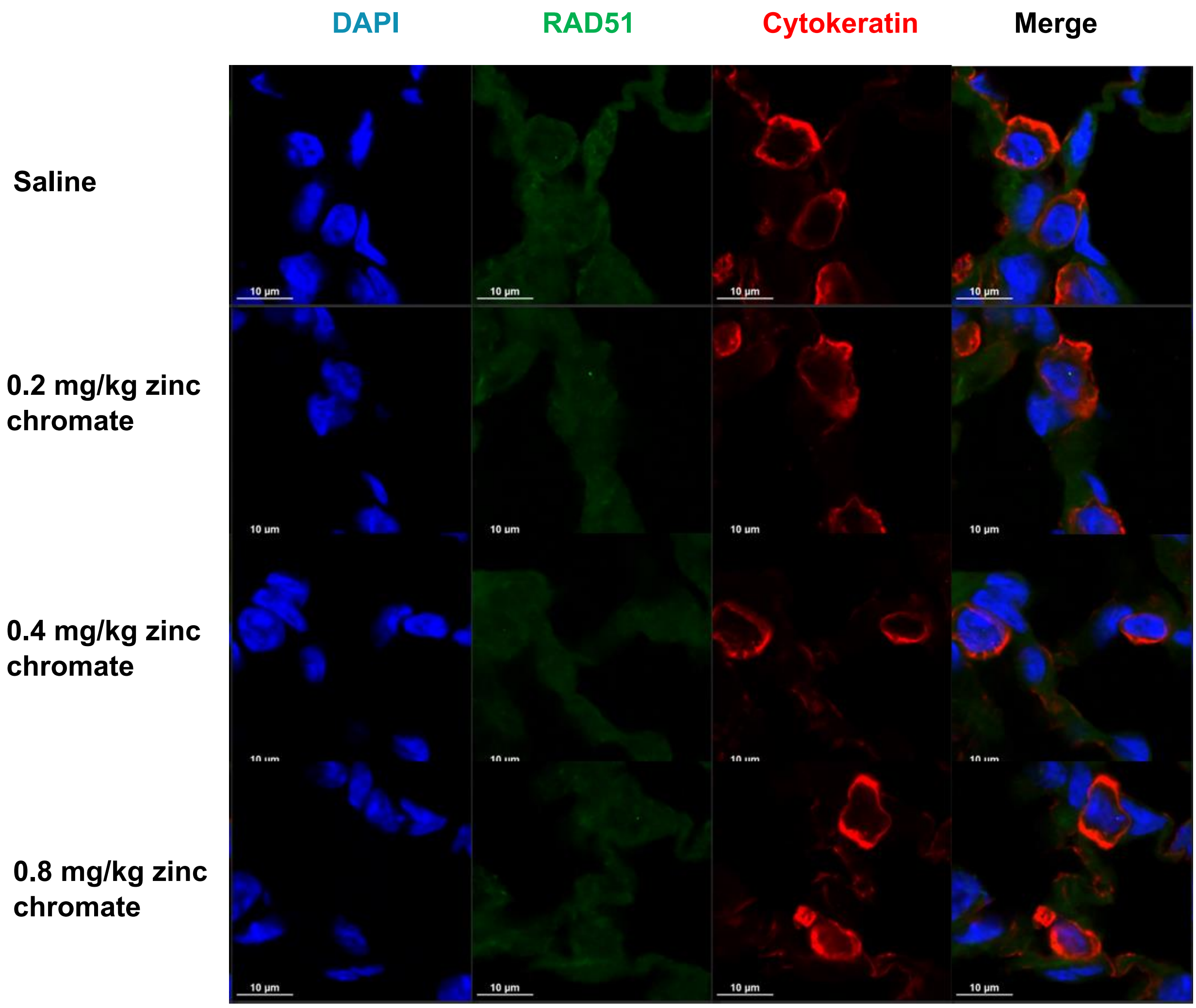

Figure S4: Representative images of RAD51 after subchronic exposure in the alveoli.

The figure shows representative images of RAD51 foci in the alveoli of guinea pigs subchronically exposed to saline or various zinc chromate concentrations. Images were obtained using a confocal microscope with a x100 objective and a x3 digital zoom. Scale bars represent 10 um. The targets are organized by column: the first column is DAPI treatment to stain the nucleus; the second column is RAD51 foci, a marker of homologous recombination repair; the third column is cytokeratin expression, a marker of epithelial cells; the fourth column is the merged image. The images were cropped and rearranged to display the merged image last.
